# Supplementary material for: Optogenetic stimulation of anterior insular cortex neurons in male rats reveals causal mechanisms underlying suppression of the default mode network by the salience network
Source: Nat Commun. 2023 Feb 16;14:866. doi: 10.1038/s41467-023-36616-8 (PMC9935890; doi:10.1038/s41467-023-36616-8)
Supplement: Supplementary file 1 — Supplementary Information [file 41467_2023_36616_MOESM1_ESM.pdf]

Optogenetic stimulation of anterior insular cortex neurons in male rats reveals causal mechanisms underlying suppression of the default mode network by the salience network

Supplementary Information

I. Supplementary Methods -- Page 2

II. Supplementary Note -- Page 6

III. Supplementary Discussion -- Page 15

IV. Supplementary Figure --- Page 22

I. Supplementary Materials and Methods

1. Bayesian Switching Dynamic Systems model

To determine latent brain state dynamics underlying optogenetic manipulation of the anterior insular cortex (AI), we used a Bayesian Switching Dynamic Systems (BSDS) model[1]. Here we briefly describe the mathematical framework of the BSDS model[1]. Let $\mathbf{y}_t^s$ denote a D-dimensional vector of region of interest (ROI) timeseries obtained from subject s in time t, where D is the number of ROIs. Following the general formulation of the switching state-space models, we defined $\mathbf{z}_t^s$ as the latent state variables and $\mathbf{x}_{kt}^s$ as the latent space variables associated to $\mathbf{y}_t^s$ at the k-th latent state, that is $z_{kt}^s = 1$. The $\mathbf{z}_t^s$ is a 1-of-K discrete vector with elements z_{kt}^s, $\forall k = 1, \dots, K$. Two successive time instances are dependent through a 1st-order Markov chain of Hidden Markov Model (HMM). Using Markovian properties and given state transition probabilities $\mathbf{A}$, where $A_{jk} \equiv p(\, z_{kt}^s = 1 | z_{j,t-1}^s = 1)$ and a marginal distribution $p(\, \mathbf{z}_1^s | \boldsymbol{\pi}) = \prod_{k=1}^K \pi_k^{z_{k1}^s}$ represented by a vector of initial probabilities $\boldsymbol{\pi}$ where $\pi_k \equiv p(z_{k1}^s = 1)$, the probability distribution for the latent state variables is expressed by $p(\, \mathbf{z}_t^s | \mathbf{z}_{t-1}^s, \mathbf{A}) = \prod_{k=1}^K \prod_{j=1}^K A_{jk}^{z_{j,t-1}^s \, z_{kt}^s}$ for all $t > 1$. We assume that at a given latent state k in time t, shown by $z_{kt}^s = 1$, the observed vector $\mathbf{y}_t^s$ is generated via probabilistic interpretation of a factor analysis model [2, 3] as:

$$\mathbf{y}_t^s = \mathbf{U}_k \mathbf{x}_{kt}^s + \boldsymbol{\mu}_k + \mathbf{e}_{kt}, \quad \forall t \, | z_{kt}^s = 1,$$

where $\mathbf{U}_k$ is a $D \times P$ dimensional linear transformation matrix that transforms data to a subspace of lower dimensionality, $P < D$, described using a P-dimensional vector of latent space variables $\mathbf{x}_{kt}^s$ mediated by an overall bias $\boldsymbol{\mu}_k$ and a measurement noise $\mathbf{e}_{kt}$. With the normality assumption, that is $\mathbf{x}_{kt}^s \sim \mathcal{N}(\mathbf{0}, \mathrm{I})$ and $\boldsymbol{e}_{kt} \sim \mathcal{N}(\mathbf{0}, \boldsymbol{\Psi}_k)$, the marginal distribution of $\mathbf{y}_t^s$ follows a Gaussian distribution as $p(\, \mathbf{y}_t^s | \boldsymbol{\mu}_k, \mathbf{U}_k, \boldsymbol{\Psi}_k) = \mathcal{N}(\boldsymbol{\mu}_k, \mathbf{U}_k {\mathbf{U}_k}^{\mathrm{T}} + \boldsymbol{\Psi}_k)$ where T denotes the transpose operator. We then define a dynamical process on the latent space variables using an autoregressive (AR) model [4] of order R as:

$$\mathbf{x}_{kt}^s = \overline{\mathbf{X}}_{kt}^s \, \overrightarrow{\mathrm{V}_k} + \boldsymbol{\varepsilon}_{kt}, \qquad \forall t \, | z_{kt}^s = 1,$$

where $\overrightarrow{\mathbf{V}_k}$ is a vector of AR coefficients. $\overline{\mathbf{X}}_{kt}^s = \mathrm{diag}(\overline{\mathbf{x}}_{kt}^s)$ is a block diagonal isotropic matrix with elements of $\overline{\mathbf{x}}_{kt}^s = \left({\overline{\mathbf{x}}_{k,t-1}^s}^{\mathrm{T}}, \; {\overline{\mathbf{x}}_{k,t-2}^s}^{\mathrm{T}}, \dots, {\overline{\mathbf{x}}_{k,t-R}^s}^{\mathrm{T}} \right)$ represented using latent space variables from the previous R time frames. $\boldsymbol{\varepsilon}_{kt} \sim \mathcal{N}(\mathbf{m}_k, \boldsymbol{\Sigma}_k)$ is the remaining error term in the latent space. All

analyses conducted in this study use a 1st-order AR model ($R = 1$). Detailed theoretical derivations are provided in the previous study [1].

2. Temporal properties of latent brain states

BSDS estimated the posterior probability of each latent brain states at each time point and chose the latent brain state with the highest probability as the dominant state at that time point. Using the temporal evolution of the latent brain states, we measured temporal properties of each latent brain state, including occupancy rate and state switching probability. Occupancy rate quantifies the proportion of time that a state is chosen as the dominant state. State switching probability quantifies the chance that brain state at time point *t* either remains at its own state or switch to another brain state at the time point *t+1*. These temporal properties were examined to characterize their relationship with optogenetic stimulation conditions.

3. Stimulation block prediction using time-varying latent brain state dynamics

Stimulation block classification analysis was performed to investigate whether time-varying latent brain state dynamics contain information associated with stimulation OFF block and stimulation ON block. We built a multiclass classifier based on a linear support vector machine using the MATLAB package LIBSVM [5] to discriminate stimulation OFF block and stimulation ON block at each time point. The posterior probabilities of the latent brain states at each time point during stimulation OFF blocks and stimulation ON blocks were used as features to train the classifier. Classifier performance was evaluated by conducting leave-one-out cross-validation (LOOCV) analysis. Specifically, posterior probability time-series of the five latent brain states from one rat were used as a test set and the posterior probability time-series of the brain states obtained from rest of the rats were used to train the classifier. Then, the trained classifier was applied to the test set to predict moment-by-moment correspondence between posterior probabilities of the latent brain states and stimulation block (OFF/ON). This procedure was performed *S* times (*S*: number of rats), and the cross-validation accuracy across the test sets was used to evaluate the performance of the classifier. We further evaluated statistical significance of LOOCV accuracy using permutation testing (500 times). In each permutation, ON and OFF block labels were randomly permuted. We followed the same LOOCV analysis procedure described above to evaluate the classification accuracy under permutation. We

repeated this procedure 500 times, and used the resulting distribution to evaluate the statistical significance of the LOOCV accuracy.

4. Regional activation and functional connectivity of latent brain states

Each latent brain state is represented by a multivariate Gaussian distribution, which is described by the mean (activation levels of ROIs) and covariance (functional connectivity between ROIs) matrices. To investigate changes in activation level of each ROI between different latent brain states, we conducted paired *t*-tests on the rat-state-wise mean values for each ROI. To determine which dynamic functional connections are important for distinguishing different latent brain states, we first computed the partial correlations from the estimated covariance matrices for each state and then conducted paired t-tests on the rat-state-wise z-transformed correlation matrices. Multiple comparisons were corrected using false discovery rates ($p < 0.05$).

5. General linear model (GLM) analysis of brain activation and connectivity associated with AI stimulation

We used a conventional general linear model (3dDeconvolve) as implemented in AFNI (ver. 20.2.10) [6] to determine brain activation associated with AI stimulation. In each rat, a regressor of interest corresponding to the ON stimulation condition was convolved with a negative monocrystalline iron oxide nanoparticle (MION) hemodynamic response function (HRF)[7]. Contrast β-maps corresponding to the stimulation ON condition were determined in each rat, and entered into a group level analysis. A one sample *t*-test (3dttest++) was used to determine group activation maps, and a voxel-wise threshold of $p < 0.005$ with family-wise cluster-correction threshold of $p < 0.01$ (cluster size = 38 voxels) was used to determine significant activation clusters.

We used a generalized psychophysiological interaction (gPPI) [8] model to determine changes in connectivity associated with AI stimulation. The gPPI model consisted of physiological terms, psychological terms, and PPI terms. The physiological terms were time-series data from the AI ROI; the psychological terms were the convolutions of the MION HRF with the main task effects of interest (ON condition timestamps); and the PPI terms were deconvolved raw time-series

data from the AI ROI multiplied by the main effect of interest followed by a convolution with the MION HRF.

II. Supplementary Note

1. Viral vector injection and optical fiber placement

We targeted the approximate center of mass of the AI for, considered to be anterior to the granular and posterior insular cortex subdivisions[9], for optogenetic stimulation. As such, fiber locations were within the dorsal and ventral agranular insular cortex (Figure 1c), although some light spread could have activated Chronos-expressing neurons in the dysgranular insular cortex as well (Figure 1b). Notably, although our fiber locations were anterior to granular and posterior insular cortex subdivisions, given the extensive reciprocal connectivity between insular cortex subdivisions [10], we cannot rule out network-level effects from these areas.

We achieved Chronos and Control vector gene expression in the right AI via high-titer (~10^{12} vg/ml) recombinant adeno-associated virus (AAV) pseudo-typed with the serotype 5 capsid and using the human synapsin (hSyn) promotor. The pan-neuronal hSyn promoter was chosen for its high transduction efficiency in cortex [11], and because further neuronal specificity was not required as the AI sends exclusively excitatory projections to other brain regions [10]. We observed virus expression throughout all layers of the AI (Figure 1b), indicating expression in both inhibitory interneuron populations and excitatory output neurons. This broad expression across cortical layers and neuron types has been reported previously for this serotype [12] and promotor [11, 12, 13]. Importantly, it has been demonstrated that optogenetic stimulation produces significantly increased multi-unit activity in rodent mPFC using the same serotype, promotor, stimulation frequency, pulse width, and a similar laser power and viral titer as employed here [14].

2. GLM-based activation and connectivity changes associated with AI stimulation

2.1. Activation: GLM analysis uncovered strong responses in the AI stimulation site in Chronos rats (Figure S2) and deactivation of the striatum and medial temporal lobe, but no response clusters in other brain regions ($p < 0.005$ voxel-wise threshold, cluster threshold $p < 0.01$). Inspection of the time-series from our a priori ROIs suggest a complex profile of temporal changes elicited by optogenetic stimulation (Figure S3). No significant clusters were detected in EYFP rats.

2.2. gPPI connectivity: gPPI connectivity analysis revealed no significant clusters ($p < 0.005$ voxel-wise threshold, cluster threshold $p < 0.01$) associated with AI stimulation in either Chronos or EYFP rats.

These results highlight the ability of latent space dynamical switching models like BSDS to capture dynamic circuit properties that are missed by conventional general linear models.

3. Latent state-specific activation and deactivation patterns in Chronos and EYFP control groups

To further expand our results to the whole-brain level, we analyzed the whole-brain maps of normalized activity for each brain state identified in the Chronos and EYFP control groups by averaging the time points assigned to each state (Figure S4a, b). For the Chronos group, this analysis demonstrated different patterns of activation and deactivation in the AI and retrosplenial cortex (RSC) across brain states. Notably, we observed activation of the RSC spanning anterior-to-middle subdivisions from -3.08 to -4.28 and deactivation of the AI in State 1, the OFF-state, and activation of the AI in State 2, the ON-state, which corroborate our results in Figure 4a. In addition, we observed deactivation of anterior-middle RSC and AI in State 3, the Transition state, which corroborates our results in Figure 6a, d, respectively.

We then computed the spatial correlation coefficients of respective states from the Chronos and EYFP groups to examine the similarity between them. The spatial patterns of State 1, the OFF-state, in the Chronos group (Figure S4a), and State 1, the primary state, in the EYFP control group (Figure S4b) showed high similarity ($r = 0.47$, Figure S4c), suggesting that the primary OFF state in the Chronos group may be the primary state in the control group. Furthermore, State 2, the ON-state, and State 3, the transition-state, in the Chronos group showed high similarity with State 3 and State 2 in the EYFP control group, respectively (Figure S4c).

4. Occupancy rates of latent brain states in AI stimulation ON and OFF sub-blocks

To further characterize stimulation-dependent changes in occupancy rates of latent brain states, we divided the stimulation OFF and ON blocks into 10 s sub-blocks (Figure S5a, b) in Chronos rats and EYFP controls, and examined occupancy rates of the brain states in each sub-block.

In Chronos rats, we found that the occupancy rate of the ON state (State 2) was significantly higher than other brain states in both sub-blocks of the stimulation ON block (Figure S5c, left, $p < 0.05$, two-tailed *t*-test, FDR-corrected), suggesting that the ON state dominates the stimulation ON block. Furthermore, the occupancy rate of the ON state (State 2) was significantly higher than other brain states in the 1st sub-block of the stimulation OFF block, which occurs immediately after the stimulation ON block (Figure S5c, right), suggesting that fMRI responses evoked by the stimulation can persist for at least 10 s. Subsequently, from the 1st to 3rd sub-block of the stimulation OFF block, the occurrence rate of the ON state progressively decreased, while the occupancy rate of State 3 and the OFF state gradually increased with time, eventually leading to sub-blocks dominated by the OFF state (Figure S5c, right). These findings suggest that State 3 may serve as an intermediate state that connects the ON and OFF states during stimulation ON-OFF boundaries and share some functional properties of the OFF state. The OFF state (State 1) dominated the remainder of the OFF block, from sub-blocks 4 through 8, suggesting that State 1 represents the latent brain state dynamic for stimulation OFF periods (Figure S5c, left, right, $p < 0.05$, two-tailed *t*-test, FDR-corrected). Next, analysis of EYFP controls revealed that a single state (i.e., State 1) dominated all the sub-blocks during the stimulation ON and OFF periods (Figure S5d, $p < 0.05$, two-tailed *t*-test, FDR-corrected), confirming that latent brain state dynamics are related to the optogenetic stimulation of AI and not induced by potential nonspecific effects of the stimulation paradigm.

Together, these analyses demonstrate that, in Chronos rats, State 1 (the OFF state) represents baseline activity, and State 2 (the ON state) represents the stimulation-evoked activity, and lastly, State 3 shares some functional similarity to the OFF state and participates as an intermediate state that connects the ON and OFF states during stimulation ON-OFF boundaries. Importantly, these results highlight the ability of latent space dynamical switching models like BSDS to capture dynamic circuit properties that would be missed by conventional GLM approaches.

5. Dynamic causal relationships between temporal dynamics of latent brain states

We examined how latent brain states influence each other. To accomplish this, we examined dynamic causal relations between time-varying posterior probabilities of the latent brain states as estimated by BSDS (Figure 3a). As noted, each latent brain state is characterized by a

unique pattern of dynamic functional connectivity that transiently links distributed brain regions; investigation of causal interactions between latent brain states could provide additional insights into dynamic brain circuit mechanisms underlying interactions between the ON and OFF states.

Analysis of causal relationships between latent brain states using multivariate dynamic state-space systems identification [15, 16, 17, 18], revealed that, during the stimulation OFF block, the OFF state had a negative causal influence on the ON state (Figure S6a, $p < 0.05$, two-tailed t-test, FDR-corrected). This pattern was reversed during the stimulation ON block, during which the ON state had a negative causal influence on the OFF state (Figure S6b, $p < 0.05$, two-tailed t-test, FDR-corrected). Furthermore, changes in posterior probability of the ON state were significantly correlated with the temporal profile of the AI response (Figure S6c, $r = 0.62$, $p = 7.1 \times 10^{-9}$, two-tailed t-test), suggesting that stimulation induces activation of the ON state and suppression of the OFF state (Figure S6d). These results further demonstrate the mutually inhibitory influence of brain states associated with AI engagement and RSC suppression.

6. Inter-regional functional connectivity using time-series data from BSDS-derived ON and OFF states

We conducted additional analyses to validate inter-regional functional connectivity estimated by the BSDS model. We used data from time-points dominated by ON and OFF states, and computed partial correlations across ROIs (Figure S7). We specifically examined stimulation-related functional connectivity changes of the AI, prelimbic cortex (PrL), and posterior RSC (-6.86 mm AP) with all other ROIs based on results shown in Figure 4d-f. For time-points dominated by the ON state, we found decreased connectivity between AI and anterior RSC (-2.90 mm AP), PrL and posterior RSC (-7.82 mm AP), and between RSC regions (-6.86 mm AP to -2.90 and -3.86 mm AP); we also found increased connectivity between PrL and cingulate cortex (Cg) (Figure S8a-c). Thus, direct estimation of functional connectivity using data from time-points corresponding to the ON and OFF states yielded convergent results and validated findings from the BSDS-estimated covariance matrices.

7. Dynamic state transition properties at stimulation boundary and OFF periods

We examined dynamic state transition properties using the BSDS-derived state-switching matrices of each rat. Because the duration of OFF-stimulation blocks is relatively long compared to the duration of the ON block, it is possible that state transition properties during OFF-stimulation blocks could smear the state transition properties at stimulation boundaries. To address this, we computed state-switching probability matrices separately for 3 time periods: (1) 60 s beginning 10 s into OFF-stimulation blocks, (2) 20 s OFF→ON stimulation boundaries beginning 10 s before ON-stimulation blocks, and (3) 20 s ON→OFF stimulation boundaries beginning 10 s before OFF-stimulation blocks (Figure S9a).

Analysis of the state switching matrix during OFF-stimulation periods revealed that State 1 (the OFF state) and State 3 have higher probabilities of switching between each other than to other states (Figure S9c, f), suggesting that State 3 may have a role as an additional OFF state. Analysis of state switching matrices for OFF→ON and ON→OFF stimulation boundaries revealed that the transition path between OFF and ON states is most likely to include State 3 ($P_{OFF \rightarrow State3} = 0.28$, $P_{State3 \rightarrow ON} = 0.20$, $P_{ON \rightarrow State3} = 0.11$, $P_{State3 \rightarrow OFF} = 0.18$, Figure S9d, e, g, h), suggesting a role for State 3 as a Transition state during stimulation boundaries. State 3 also has the second-highest occupancy rate during OFF-stimulation blocks (Figure S9b), consistent with the observation that this state functions as both a secondary OFF state and an intermediate state between ON and OFF states.

Lastly, examination of temporal properties of State 4 and 5 showed that they not only have low occurrence during stimulation boundaries, but also do not have distinct patterns in their occurrence, suggesting that these two states do not function as Transition or OFF states.

8. Replication of dynamic functional connectivity changes during the Transition compared to the ON and OFF states

We extended the above analyses to validate inter-regional functional connectivity during the Transition compared to the ON and OFF states estimated by the BSDS model. We used data from time-points dominated by Transition, ON and OFF states, and computed partial correlations across ROIs (Figure S7). We then compared the results with findings from state covariance matrices driven directly by BSDS (Figure 6c, f). The two approaches for estimating connectivity changes between Transition and OFF state showed convergent results.

Specifically, for time-points dominated by the Transition, compared to the OFF, state we found stronger functional connectivity between the AI and mid-RSC (-5.90 mm AP), but reduced intra-RSC connectivity between anterior (-2.90 and –3.86 mm AP) and mid-posterior subdivisions of RSC (-5.90 mm, -6.86 mm AP) (Figure S10a, all $ps < 0.05$, two-tailed t-test, FDR corrected).

Similarly, the two approaches also showed convergent results for connectivity changes between the ON and Transition states. The ON state showed increased connectivity between the PrL and Cg, but decreased connectivity between anterior-middle RSC (-3.86 mm, -4.86 mm AP) and posterior RSC subdivisions (-6.86 mm, -7.82 mm AP) (Figure S10b, all $ps < 0.05$, two-tailed *t*-test, FDR-corrected). Thus, direct estimation of functional connectivity using data from time-points corresponding to the Transition, ON and OFF states yielded convergent results and validated findings from the BSDS-estimated covariance matrices.

9. Replication of findings using extended salience network (SN) and default mode network (DMN) ROIs

We examined the robustness of our findings with respect to ROI selection with additional subcortical nodes. We conducted additional analysis by incorporating hippocampus and amygdala nodes, which are known to be part of SN [19] and DMN [20], respectively (Figure S11a). As described below, all major findings were replicated with this new set of ROIs.

9.1. Matching BSDS states estimated from different ROI sets: BSDS identified 3 latent brain states. To determine whether brain states identified in the original model matched brain states identified in the extended ROI set, we conducted cross-model brain state correlation analysis. By computing Pearson's correlation of posterior probabilities of latent brain states estimated from the two distinct models, we found high one-to-one mapping between $S1_{original}$ and $S1_{extended}$, between $S2_{original}$ and $S2_{extended}$, and between $S3_{original}$ and $S3_{extended}$, respectively (Figure S11b). These supplementary results demonstrate robustness of our main finding that distinct latent brain state dynamics dominate stimulation OFF and ON blocks, as well as their boundaries.

9.2. Spatiotemporal properties of extended DMN and SN ROI-derived latent brain states corresponding to stimulation ON and OFF blocks: Each latent brain state showed distinct moment-by-moment changes in posterior probability across stimulation ON and OFF blocks (Figure S11c). Examination of AI stimulation effects on the temporal properties of each state

revealed that State 1 has a significantly higher occupancy rate than other states during stimulation OFF blocks (Figure S11d, all *ps* < 0.05, two-tailed *t*-test, FDR-corrected). Furthermore, the occupancy rate of State 1 was significantly higher during stimulation OFF blocks compared to stimulation ON blocks (Figure S11d, $p < 0.05$, two-tailed *t*-test, FDR-corrected), suggesting that State 1 is the primary state associated with the stimulation OFF blocks (OFF state). In contrast, State 2 had a significantly higher occupancy rate than other states during stimulation ON blocks (Figure S11d, all *ps* < 0.05, two-tailed *t*-test, FDR-corrected). Furthermore, the occupancy rate of State 2 was significantly higher during stimulation ON compared to stimulation OFF blocks (Figure S11d, $p < 0.05$, two-tailed *t*-test, FDR-corrected), implying that State 2 is a dominant state associated with the stimulation ON block (ON state).

We next examined dynamic functional connectivity associated with the ON and OFF brain states. Univariate link-specific analysis was conducted to determine unique functional connectivity patterns that differentiate the ON and OFF states (Figure S11e). This analysis revealed that the ON state has unique connectivity patterns compared to the OFF state (all *ps* < 0.01, two-tailed *t*-test, FDR-corrected). Notably, the ON state showed decreased connectivity between AI and an anterior RSC subdivision (-2.90 mm AP), suggesting decoupling between SN and DMN. In addition, the ON state showed increased connectivity between the PrL and Cg, and the decreased connectivity between the PrL and posterior RSC subdivisions (-6.86 mm, -7.82 mm AP) and between anterior and posterior RSC subdivisions. Furthermore, the ON state also showed increased connectivity between the amygdala and PrL, AI, and Cg. These results suggest functional involvement of the PrL, Cg, and amygdala in SN, and functional heterogeneity within the RSC underlying SN-DMN dynamics.

Taken together, these results are consistent with our main findings, highlighting the robustness of our findings.

10. Replication of findings using SN and DMN nodes and additional striatum-MTL ROIs

We further examined the robustness of our findings with respect to ROI selection by including nodes for the most significant response clusters detected by GLM (Figure S2), the striatum and the medial temporal lobe (MTL) (Figure S12a). Although the involvement of striatum and MTL is not as well established within large-scale resting-state networks in the rodent brain as our *a*

priori ROIs, they are generally considered part of SN [21, 22] and DMN [23, 24, 25], respectively. As described in detail below, all major findings were replicated with this new set of ROIs.

10.1. Matching BSDS states estimated from different ROI sets: BSDS identified four latent brain states. To determine whether brain states identified in the original model matched brain states identified in the extended ROI set, we conducted cross-model brain state correlation analysis. By computing Pearson's correlation of posterior probabilities of latent brain states estimated from the two distinct models, we found high one-to-one mapping between $S1_{original}$ and $S1_{extended}$, between $S2_{original}$ and $S2_{extended}$, and between $S3_{original}$ and $S3_{extended}$, respectively (Figure S12b). These supplementary results demonstrate robustness of our main finding that distinct latent brain state dynamics are associated with optogenetic stimulation of the AI.

10.2. Spatiotemporal properties of extended ROI-derived latent brain states corresponding to stimulation ON and OFF blocks: Each latent brain state showed distinct moment-by-moment changes in posterior probability across stimulation ON and OFF blocks (Figure S12c). Examination of AI stimulation effects on the temporal properties of each state revealed that State 1 has a significantly higher occupancy rate than other states during stimulation OFF blocks (Figure S12d, all $ps < 0.05$, two-tailed t-test, FDR-corrected). Furthermore, the occupancy rate of State 1 was significantly higher during stimulation OFF blocks compared to stimulation ON blocks (Figure S12d, $p < 0.05$, two-tailed t-test, FDR-corrected), suggesting that State 1 is the primary state associated with the stimulation OFF blocks (i.e., OFF state). In contrast, State 2 had a significantly higher occupancy rate than other states during stimulation ON blocks (Figure S12d, all $ps < 0.05$, two-tailed t-test, FDR-corrected). Furthermore, the occupancy rate of State 2 was significantly higher during stimulation ON compared to stimulation OFF blocks (Figure S12d, $p < 0.05$, two-tailed t-test, FDR-corrected), implying that State 2 is a dominant state associated with the stimulation ON blocks (i.e., ON state).

We next examined dynamic functional connectivity associated with the ON and OFF brain states. Univariate link-specific analysis was conducted to determine unique functional connectivity patterns that differentiate the ON and OFF states (Figure S12e). This analysis revealed that the ON state has unique connectivity patterns compared to the OFF state (all $ps < 0.05$, two-tailed t-test, FDR-corrected). Notably, the ON state showed decreased connectivity of the AI with anterior RSC subdivision (-2.90 mm AP), Striatum and MTL. In addition, the ON

state showed increased connectivity between the PrL and Cg, and decreased connectivity between the PrL and posterior RSC subdivisions (-6.86 mm, -7.82 mm AP) and between anterior and posterior RSC subdivisions. These results suggest that the striatum and MTL are involved in SN-DMN dynamics, and are overall consistent with our main results, highlighting the robustness of our findings.

11. Control analysis using ROIs located outside the SN and DMN

We conducted additional control analyses using ROIs from the auditory, visual, and motor cortex outside of the canonical SN and DMN (**Figure S13a**) to investigate the specificity of our findings with respect to SN and DMN nodes. BSDS identified 4 latent brain states from the new ROI set. In contrast to our main analysis, latent brain states estimated from the new ROI set did not show stimulation-dependent changes in posterior probability (**Figure S13b**). Furthermore, examination of the occupancy rate of each latent brain state during stimulation OFF and ON blocks demonstrated that the major findings reported in our main analyses (i.e., stimulation-dependent changes in occupancy rate of latent brain states) are not observed with the new ROI set (**Figure S13c**). Taken together, these results demonstrate the specificity of our findings with respect to canonical SN-DMN ROIs.

III. Supplementary Discussion

1. Rationale for selectively targeting right AI rather than left AI

Our choice of targeting the right AI is based on previous findings about lateralization of AI dynamic causal influence on network switching [26]. Specifically, recent dynamic causal analysis of human fMRI data indicated that the right AI has significantly higher net causal outflow than left AI and is likely to be responsible for dynamic network switching between SN and DMN in healthy subjects [16]. In agreement, damage to the right AI in disease states impairs this dynamic network interaction [26, 27, 28, 29]. Based on these findings, and potential for translational neuroscience research, we targeted the right AI and investigated the causal influence of AI stimulation on putative DMN nodes. We predict similar effects with left AI stimulation in rodents, since the rodent brain shows less hemispheric specialization [30].

2. Key advantages of BSDS over other approaches

BSDS belongs to a class of latent space switching models [31, 32] which goes beyond conventional methods, and has been shown to be highly effective in recovering the structure of non-stationary time-varying organization of neural circuits [1, 33, 34]. Our computational model-based approach, BSDS[1], has the following key advantages over other approaches:

1. First, BSDS does not require arbitrary sliding windows nor does it impose temporal boundaries associated with predefined task conditions - this is contrast to previous approaches for characterizing dynamic interactions in the brain that have primarily been based on sliding window or clustering techniques (e.g., ICA or PCA-based approaches) applied to observed fMRI data[35, 36, 37]. These previous methods rely on ad hoc procedures for determining critical parameters, such as the window length and number of brain states (i.e., clusters), which are known to greatly influence the estimation of dynamic brain states and connectivity [38]. In contrast, BSDS uses a Bayesian framework to automatically regulate model complexity and directly estimates the optimal number of latent states.
2. Second, BSDS applies a hidden Markov model (HMM) to latent space variables of the observed fMRI data, resulting in a parsimonious model of generators underlying the observed data – whereas previous approaches have applied hidden Markov models directly to observed fMRI data without estimating the underlying latent states [39, 40, 41, 42]. Instead, BSDS applies HMM to latent space variables generated by an autoregressive process,

resulting in greater robustness to abrupt and noisy local changes in the observed data and more robust state identification.

3. Third, BSDS allows us to uncover 'latent' brain states and their dynamic spatiotemporal properties, including probability and sequence of state transitions as well as inter-regional functional connectivity associated with each brain state, in an optimal latent subspace. In our previous study (Taghia et al[1]), we demonstrated that BSDS has greater robustness to the abrupt noisy and local changes in fMRI, resulting in more accurately identifying brain states and their temporal dynamic properties than conventional data driven approaches (e.g., ICA or PCA-based approaches). Based on these advantages, we used BSDS in our study to investigate latent brain state dynamics underlying optogenetic manipulation of the AI.

Taken together, our analyses demonstrate that BSDS can capture dynamic circuit mechanisms of optogenetic stimulation that are missed by conventional analyses.

3. Rationale for anatomically-defined SN and DMN ROIs

Because the focus of our study was to investigate AI stimulation-induced changes in SN-DMN interactions in rodent brain, we selected AI, PrL, Cg, and RSC as the most logical ROIs to conduct the analyses based on a wide range of published studies [19, 20, 22, 43, 44, 45, 46, 47, 48, 49, 50]. Because conventional GLM analyses did not uncover strong responses in these ROIs, except for the AI stimulation site (Figures S2 and S3), we used anatomically-defined canonical SN and DMN nodes encompassing the AI, PrL, Cg, and RSC [19, 20, 22, 43, 44, 45, 46, 47, 48, 49, 50].

Our use of anatomically-defined ROIs was also motivated by inconsistencies in identification of SN and DMN nodes in resting-state fMRI studies. While analysis of functional connectivity using resting-state fMRI has identified a rodent DMN anchored in the RSC [19, 20], there is less agreement about inclusion of medial prefrontal cortex DMN nodes, such as the Cg and PrL [20, 44, 46, 47, 49, 50]. Indeed, in a recent study characterizing the rodent SN, Cg and PrL were considered as key nodes of the SN in addition to their putative roles in the DMN [19]. Recent imaging studies have also paradoxically assigned individual subdivisions of the RSC and medial prefrontal cortex to both the SN and DMN [19, 22, 45, 51, 52, 53, 54]. Furthermore, the RSC is one of the largest cortical regions in rodents, and there is growing evidence for functional heterogeneity along its anterior/posterior (A/P) axis [43, 52] . Based on the divergent reports in the literature, and to more

accurately identify and model SN-DMN functional interactions, we directly probed the role of AI, Cg, PrL and multiple RSC subdivisions and their dynamic interactions during stimulation of the AI node of the SN.

4. BSDS model-based estimation illustrates the power of latent space models for capturing dynamic circuit mechanisms of optogenetic stimulation

Our analyses demonstrate that BSDS model-based estimation captures dynamic circuit mechanisms of optogenetic stimulation that are missed by conventional GLM analyses. First, conventional GLM analysis where stimulation ON and OFF blocks were directly contrasted uncovered significant response clusters in the AI, striatum, and MTL, but not in other SN and DMN nodes (Figure S2). Second, gPPI analysis did not uncover significant changes in AI connectivity at the whole-brain level. Third, inspection of time-series from SN and DMN nodes revealed that the shortcomings of GLM-based approaches may be due to the complex and delayed temporal profile of fMRI signal changes elicited by optogenetic stimulation (Figure S3). Fourth, analysis of stimulation OFF sub-blocks following the stimulation ON block revealed delayed transition patterns (Figure S5) which illustrate why conventional approaches may not accurately estimate stimulation-dependent spatiotemporal changes in brain activity and connectivity. Importantly, our results highlight the importance of characterizing intermediate transition states connecting states related to stimulation ON and OFF blocks. Taken together, these results highlight the ability of latent space dynamical switching models like BSDS to identify hidden latent brain states and their spatiotemporal dynamics underlying the effects of stimulation that are missed by conventional approaches.

5. Antagonistic functional relationship between the SN and DMN

Our findings demonstrate that AI activation causally suppresses DMN activity and is one likely mechanism underlying the functional antagonistic role between the SN and DMN networks. This, however, does not rule out other mechanisms including the possibility that the DMN activation might also directly, perhaps subsequently, suppress SN activity. Although no direct evidence exists yet, we do think that optogenetic inhibition of the AI might be sufficient to induce an activation of the DMN. Characterizing the differential causal role of SN and DMN nodes in this regard is an important goal for future studies.

6. Heterogeneity of PrL network function

There is functional and anatomical evidence that rodents do not have a dorsolateral PFC analogous to primates [55]; instead, many dorsolateral PFC functions, such as working memory, are features of the primordial rodent mPFC/PrL [56]. The rat PrL spans from approximately 2.5 mm to 5 mm AP relative to Bregma [57]. While most studies of rodent PrL in working memory have focused on the PrL AP mid-point [56, 58, 59, 60], also used for the PrL ROI here (~3.2 mm AP), there is some evidence that working memory is most susceptible to noradrenergic [59] and dopaminergic [61] manipulations of the anterior and posterior PrL, respectively, hinting at an anatomical gradient in PrL working memory function. However, other studies have demonstrated that neurons across the entirety of rat PrL encode working memory [62] and that pyramidal neurons at the posterior-most coordinates of PrL in mouse are also critical for working memory [63]. Given this mixed evidence, it is possible that the conflicting evidence here is also caused by an anatomical/functional heterogeneity. Critically, in the context of the present study, Lu and colleagues [25] suggest that the entire medial ridge (including the AP extent of PrL) is involved in the rat DMN, while Tsai and colleagues [19] show the AP extent of PrL involved in the rat SN. Taken together, dynamic functional heterogeneity, as well as anatomical and neurochemical along the AP axis of PrL, may contribute to its variable association with the DMN and SN. More precise evidence for integration rather than segregation of DMN and SN within the rodent mPFC, supported by distinct mPFC neuron populations, may be obtained by future studies employing spatially resolved recordings of neuronal activity.

7. Correspondence with studies of deep-brain structures implicated in DMN regulation

Recent studies have implicated deep-brain structures with long-range, widespread projections in DMN function and regulation (see Aguilar et al., [64] for a comprehensive review). For example, in rats, Nair et al., 2018 found that spontaneous, basal forebrain (BF), gamma-band activity was elevated during DMN-related behaviors and suppressed during other activities [65]. Subsequent rodent studies employing targeted BF stimulation or inhibition have shown that this structure can regulate the DMN. As such, nonspecific BF stimulation and stimulation of GABAergic BF projections at gamma frequencies enhances DMN-related behavioral and electrophysiological phenotypes and suppresses SN-related behaviors [66, 67], whereas BF inhibition has the opposite effects [66], and inhibition of GABAergic BF neurons can reduce ketamine-induced, DMN-related, cortical gamma band power [68]. Tonic stimulation of GABAergic BF neurons[68] or suspected

disinhibition of the BF by inhibition of somatostatin-positive BF neurons [69, 70] produces a complex phenotype suggestive of a loss of DMN regulation and cortical hyper-excitability. While the aforementioned studies focused on specific anterior DMN nodes and point to the involvement of BF GABAergic transmission, Peeters et. al., 2020 found a global reduction in DMN functional-connectivity as a result of BF cholinergic stimulation during fMRI [71], indicating a potentially cholinergic-specific role of the BF in DMN modulation. In addition, we have previously shown that tonic activation of locus coeruleus (LC) adrenergic projections decreases activity but increases functional connectivity within the anterior DMN and strengthens anticorrelated coupling between the anterior DMN and the AI [72] — thus, this system could also promote DMN-SN network switching. Notably, both the BF [73] and LC [74] are reciprocally connected with the AI; therefor, although the specific functions of these connections have yet to be investigated, it is plausible that deep-brain regulation of the DMN includes engagement and/or modulation of the AI-centered effects and SN-DMN interactions elucidated in the present study.

8. Heterogeneous activation and functional coupling of PrL and Cg during ON and OFF states

In this study, we observed significant activation of PrL, as well as increased functional connectivity between PrL and Cg but no significant activation of Cg in the ON state compared to the OFF state. While it is generally assumed that increased functional connectivity between two regions requires a concurrent increase or decrease in activity in both regions, it is not a necessary condition. An instantaneous temporal pattern of a region could vary without a change in average power, resulting in changing functional connectivity (i.e., correlation) with other regions. For instance, Krieger-Redwood et al. [75], reported increased coupling of posterior cingulate cortex (pCC) with prefrontal regions despite pCC deactivation and prefrontal activation during demanding tasks, demonstrating a heterogeneous relationship between activation profiles and functional coupling. Indeed, in a recent study characterizing the rodent SN, Cg and PrL were implicated as nodes of the SN in addition to their putative roles in the DMN [19]. We believe our results demonstrate functional heterogeneity of these two regions, which is an important research question to be addressed in more in detail in future studies.

9. Circuit-level explanation for RSC suppression by AI stimulation given current anatomical evidence

At least four parsimonious, non-mutually exclusive, circuit-level explanations could account for RSC inhibition by stimulation of excitatory AI output. (1) AI could send direct projections to inhibitory interneurons in RSC which can inhibit local principal neurons via feedforward inhibition [76], but robust AI-RSC projections have not been shown in the tract tracing literature. (2) AI output could enhance activity of intermediary nodes (e.g. PrL or Cg) that in turn send excitatory projections to RSC [77], and these projections could preferentially drive RSC inhibitory interneurons, or (3) AI output could preferentially target inhibitory interneurons in intermediate nodes, thereby reducing excitatory drive from these nodes to RSC principal neurons; however, the cell-type specific targets of AI to intermediary node projections or intermediary node to RSC projections have not yet been characterized. (4) RSC subdivisions share dense reciprocal connections [78, 79, 80], and if these connections preferentially target interneurons, then activation of one subdivision as a direct or indirect result of AI stimulation could inhibit other subdivisions. While this explanation could also contribute to RSC heterogeneity [81], described in the literature and corroborated by our findings, RSC heterogeneity could be accounted for by differential input from other brain areas. Further anatomical and neural dissection of these putative pathways should reveal critical anatomical circuit-level interactions underlying SN-DMN network interactions.

10. Potential effects of anesthesia and importance of replication experiments in awake animals

In this study, we employed a well-established, low-dose, dexmedetomidine/isoflurane anesthesia protocol [82, 83, 84, 85] to optogenetically interrogate AI activation effects with minimal contamination from background neuronal activity or DMN suppression by salient stimuli. Although static FC networks show good correspondence between this protocol and awake conditions [86, 87], and stimulus-evoked responses are robust [84], more profound differences have been observed in dynamic FC [88], especially for networks related to complex behaviors, attention, and cognitive function like the DMN and SN [89]. In addition, the present findings were acquired from male rats only because sex-specific differences are frequently reported in rodent responses to anesthesia [90, 91, 92, 93], and these differences have yet to be characterized for the aforementioned anesthetic protocol. Critically, sex differences in FC within and between the

human DMN and SN have been associated with studies divergent outcomes in normal and aberrant development [94, 95], aging [96, 97, 98], and other aspects of cognitive function [99, 100, 101, 102]. Further work with awake animals is needed to validate and replicate our findings and rigorously investigate sex-specific differences in dynamic causal network interactions.

11. Correspondence with the study of Mandino and colleagues [22]

The spatial pattern of fMRI signal changes to optogenetic AI stimulation observed in our study is corroborated by recently reported findings from optogenetic AI stimulation in mice by Mandino and colleagues [22]. Although differences between species (rats vs mice) and measurement modality (cerebral blood volume (CBV) vs blood-oxygen-level-dependent (BOLD) signal) limit direct comparisons, the response areas from our GLM analysis approximate the most significant response areas reported by Mandino et al. [22], including: the AI and cortex dorsomedial to AI, a band extending from M1 through the ventral striatum, and an anterior-ventrolateral portion of MTL (Figure S2). In addition, the signal profiles from individual ROIs in our study allude to responses in other locations similar to those shown by Mandino et. al. [22], such as PrL and RSC at -5.90 mm AP (Figure S3). Notably, CBV signal has been shown to be spatially [103] and functionally [104] more specific to neuronal activity than BOLD signal. Furthermore, Mandino et al. [22] used a considerably stronger stimulation than in the present study, including a larger bolus of optogenetic viral vector and greater stimulation light power and pulse duration despite the smaller relative volume of the mouse brain. Therefore, differences in the overall response area and regional response amplitude outside of AI could be partially explained by the relative intensity of AI stimulation between studies. In addition, as diversity in AI structural and functional connectivity has been reported along the anterior-posterior [10] and dorsal-ventral axis [19], the larger response area observed by Mandino et. al. [22] compared to the present study can also be attributed, in part, to a larger area recruited by stimulation. Nonetheless, the overall agreement between these findings provides validation for our optogenetic manipulation of AI, and points to considerable overlap in AI functional connectivity between rats and mice.

IV. Supplementary Figures

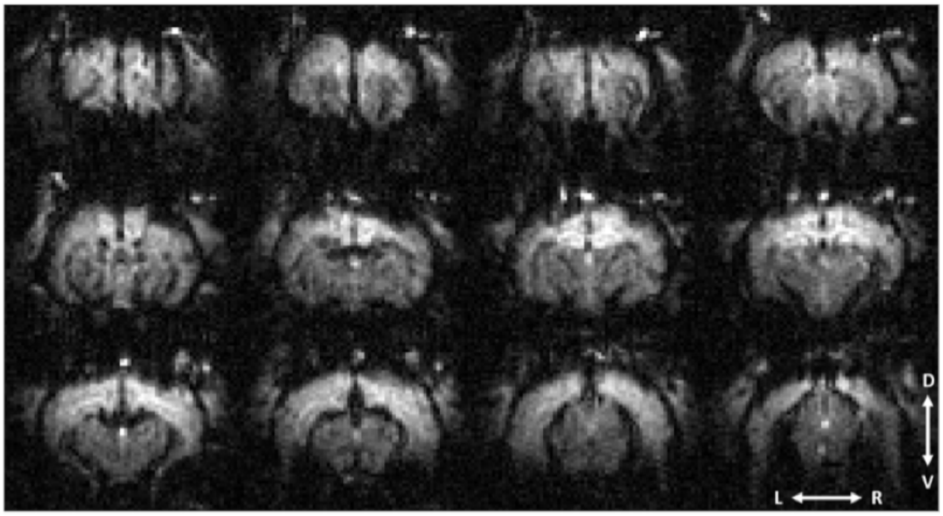

Figure S1. Representative image volume from raw, cerebral blood volume (CBV)-contrast-enhanced echo planar imaging (EPI) data. Data correspond to the first volume of the anterior insular cortex (AI)-stimulation fMRI scan for a Chronos rat. Coronal slices are displayed posterior to anterior, from top-left to bottom-right. D = dorsal, V = ventral, L = left, R = right.

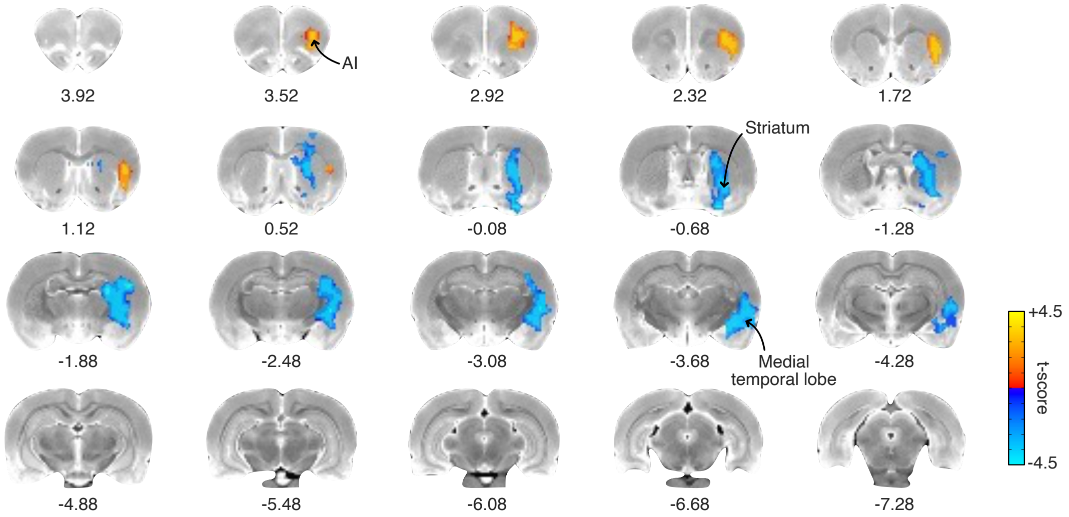

Figure S2. General linear model-based activation changes induced by optogenetic anterior insular cortex (AI) stimulation in Chronos rats. AI stimulation increased signals in the AI, striatum, and medial temporal lobe (two-tailed, one-sample *t*-test; $p < 0.005$ voxel-wise threshold; $p < 0.01$, 38 voxel cluster size, family-wise cluster-correction threshold; $n = 9$ rats).

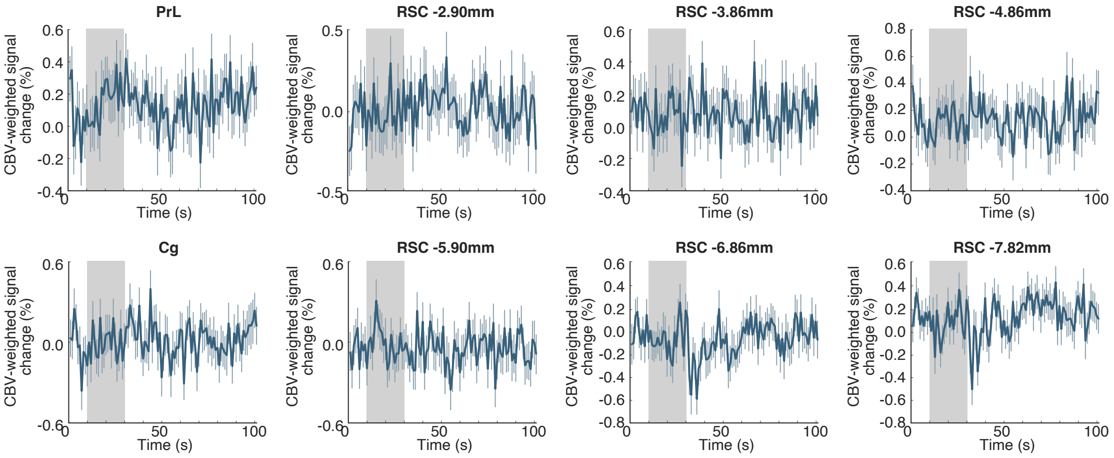

Figure S3. Evoked responses in regions downstream from optogenetic stimulation of anterior insular cortex (AI). Averaged response of prelimbic cortex (PrL), cingulate cortex (Cg), and subdivisions of retrosplenial cortex (RSC) to optogenetic AI stimulation in Chronos rats. Cerebral blood volume (CBV)-weighted signal change data are presented as mean ± SEM (*n* = 9 rats). Source data are provided as a Source Data file.

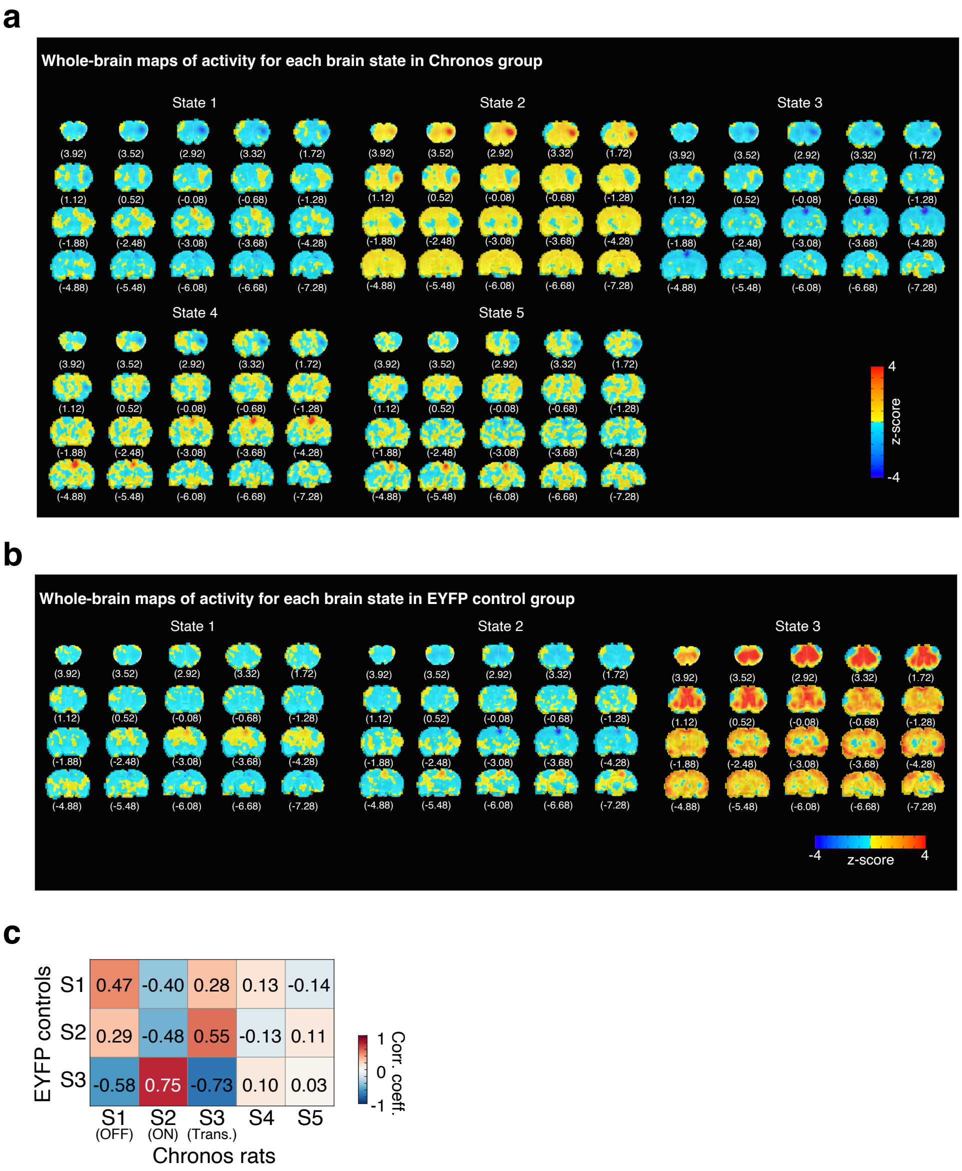

Figure S4. Whole-brain maps of activity in each brain state in the Chronos and EYFP control groups. **(a)** Chronos group (*n* = 9 rats) and **(b)** EYFP control group (*n* = 7 rats). Activation/deactivation maps were derived for each brain state by averaging time points assigned to each state. **(c)** Spatial correlation between brain states in Chronos and EYFP control groups.

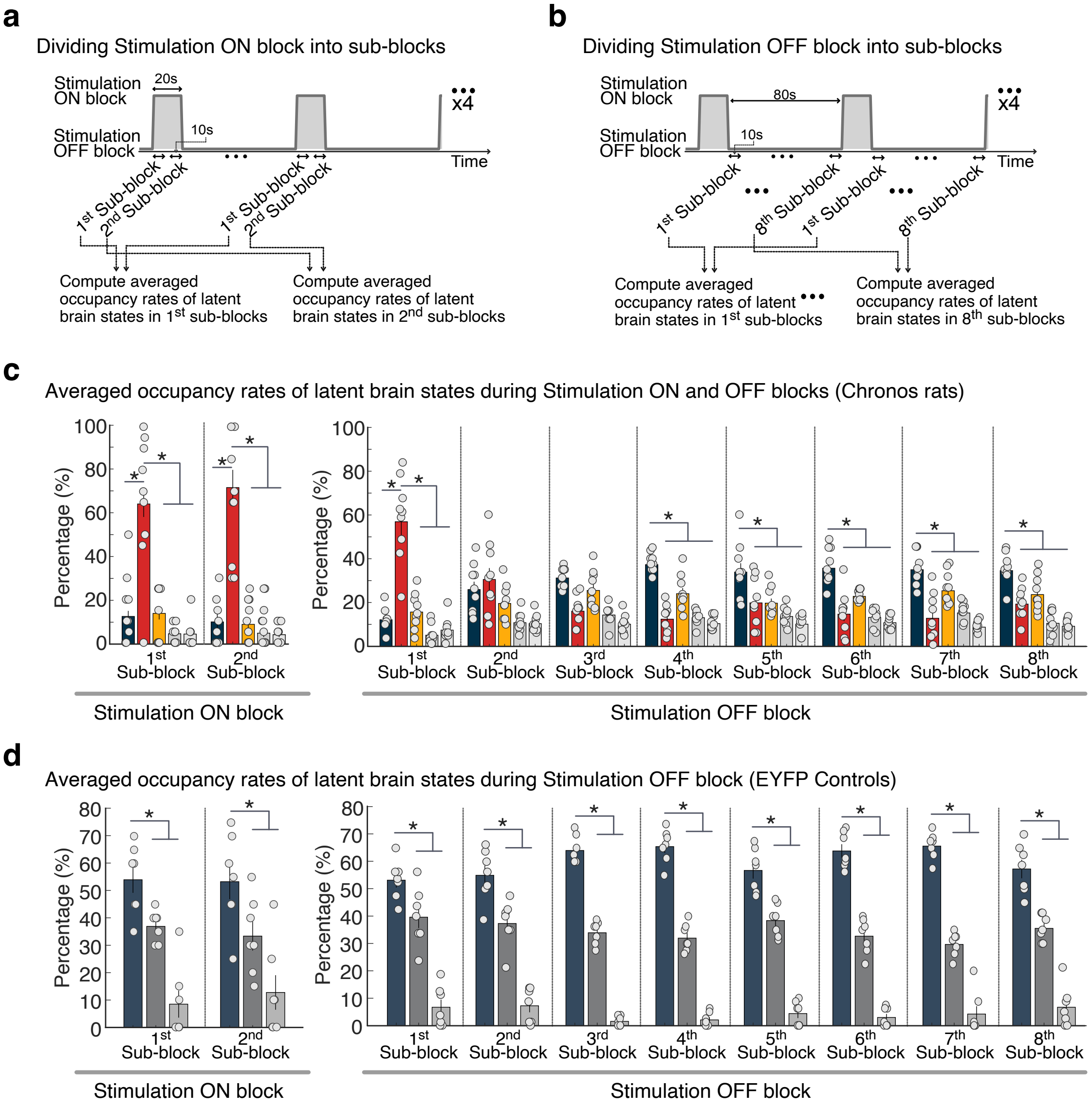

Figure S5. Occupancy rates of latent brain states during stimulation ON and OFF blocks. **(a, b)** Optogenetic stimulation protocol. We divided stimulation ON and OFF blocks into 10 s sub-blocks, respectively. **(c)** Averaged occupancy rates of latent brain states during each stimulation ON and OFF sub-block in Chronos rats ($n = 9$ rats). **(d)** Averaged occupancy rates of latent brain states during each stimulation ON and OFF sub-block in EYFP controls ($n = 7$ rats). **(c, d)** Data are presented as mean $\pm$ SEM; * $p < 0.05$, p-values determined by two-tailed t-test, with FDR-correction; all exact p-values are provided in Source Data. Source data are provided as a Source Data file.

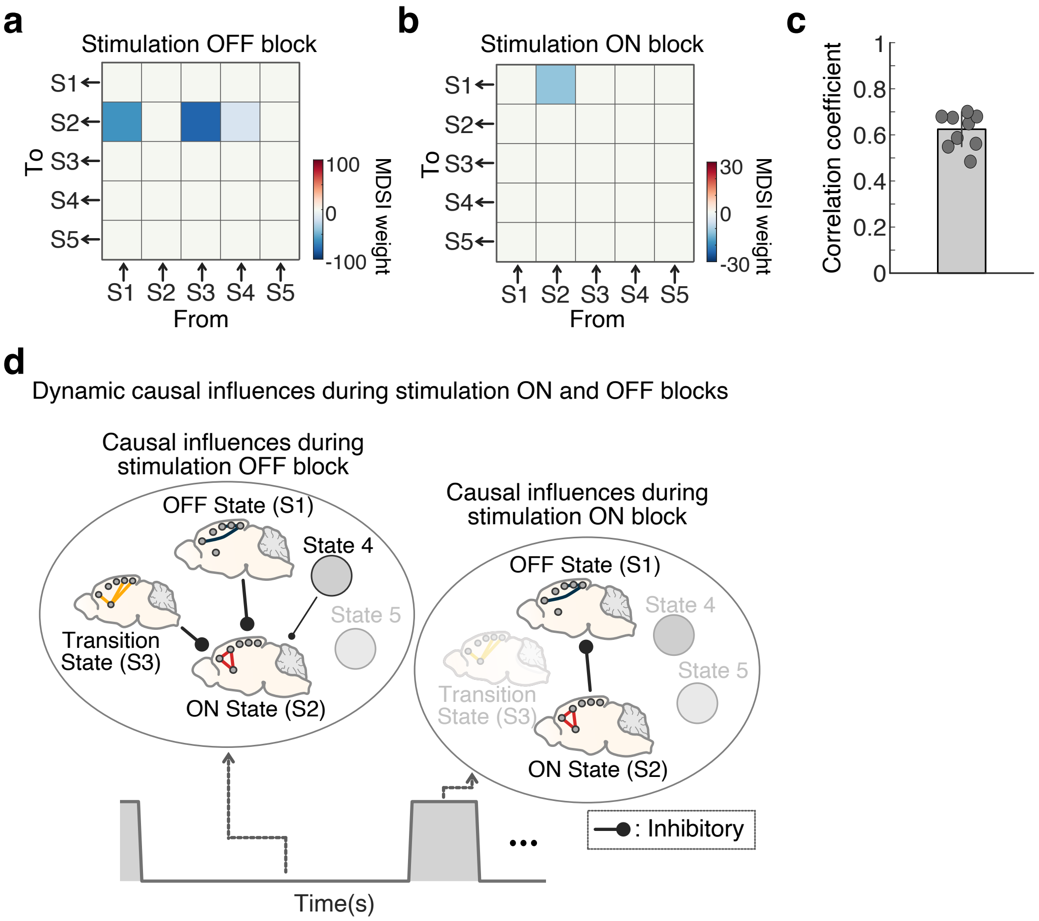

Figure S6. Dynamic causal relationships between temporal dynamics of latent brain states (a, b) Significant causal influences between latent brain states during the stimulation OFF and ON blocks ($p < 0.05$, two-tailed t-test, FDR-corrected, $n = 9$ rats; all exact p-values are provided in Source Data). Red cells indicate positive influences (i.e., activation) and blue cells indicate negative influences (i.e., inhibition) discovered by multivariate dynamic state-space systems identification (MDSI) analysis. **(c)** Correlation between the anterior insular cortex (AI) fMRI response and the temporal profile of posterior probability of State 2 (i.e., ON state). Data are presented as mean ± SEM ($n = 9$ rats). **(d)** Illustration showing dynamic causal interactions between latent brain states during the stimulation OFF and ON blocks. Source data are provided as a Source Data file.

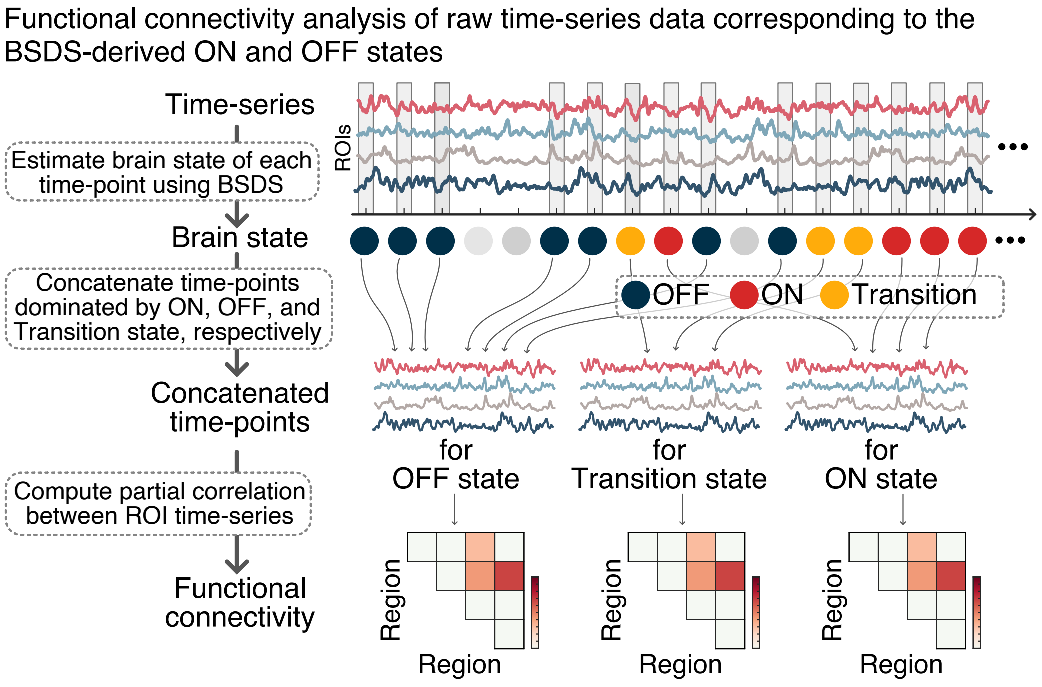

Figure S7. Key steps in functional connectivity analysis of region of interest (ROI) time-series data corresponding to Bayesian switching dynamical systems (BSDS)-derived ON and OFF states.

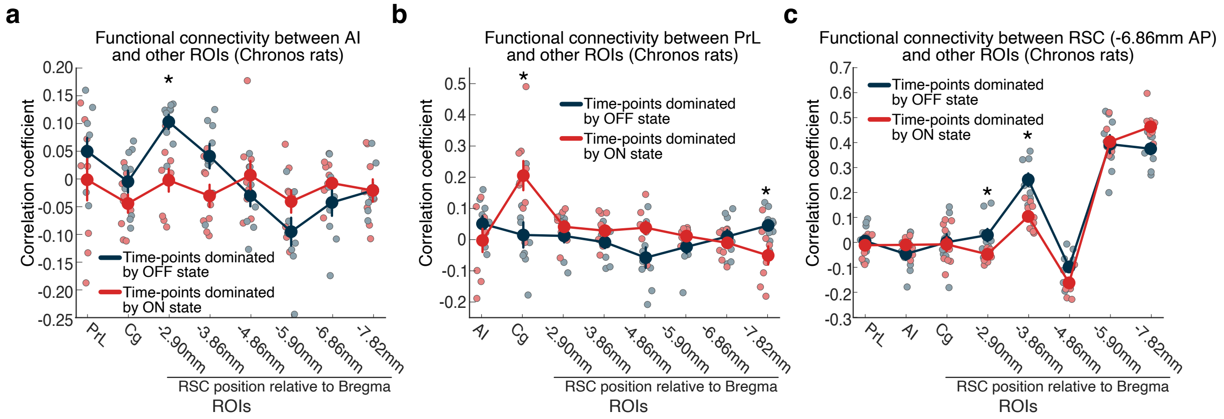

Figure S8. Inter-regional functional connectivity using time-series data from Bayesian switching dynamical systems (BSDS)-derived ON and OFF states. (a-c) Functional connectivity changes between anterior insular cortex (AI), prelimbic cortex (PrL), and posterior retrosplenial cortex (RSC; -6.86mm AP) and all the other regions of interest (ROIs) induced by optogenetic stimulation of the AI in Chronos rats. Abbreviations: cingulate cortex (Cg). These results converge on and validate functional connectivity changes reported in Figures 4d-f. Data are presented as mean ± SEM ($n = 9$ rats). *$p < 0.05$, p-values determined by two-tailed t-test, FDR-corrected; all exact p-values are provided in Source Data. Source data are provided as a Source Data file.

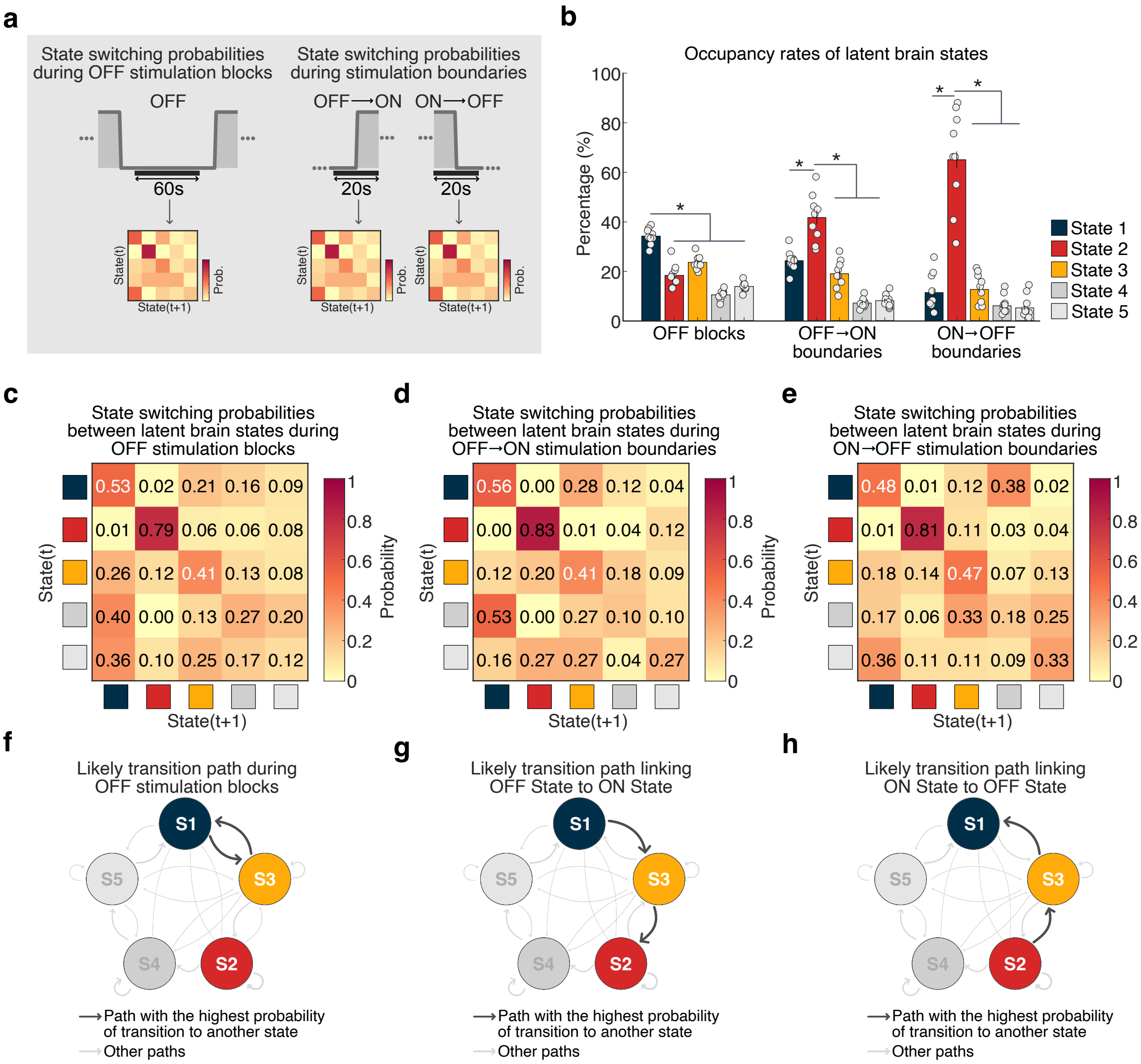

Figure S9. State switching properties of State 3 during OFF stimulation blocks and stimulation boundaries. (a) Schematic illustrating examination of state switching matrices during different periods of the experiment. **(b)** Occupancy rates of latent brain states during OFF stimulation blocks and stimulation boundaries. Data are presented as mean ± SEM (n = 9 rats). $^*p < 0.05$, p-values determined by two-tailed t-test, FDR-corrected; all exact p-values are provided in Source Data. **(c-e)** State switching matrices identified state transitions during OFF stimulation blocks, OFF→ON stimulation boundaries and ON→OFF stimulation boundaries, respectively. **(f-h)** Likely transition path during OFF stimulation blocks, OFF→ON stimulation boundaries and ON→OFF stimulation boundaries, respectively, based on the state switching probabilities between states in panel **c-e**. Source data are provided as a Source Data file.

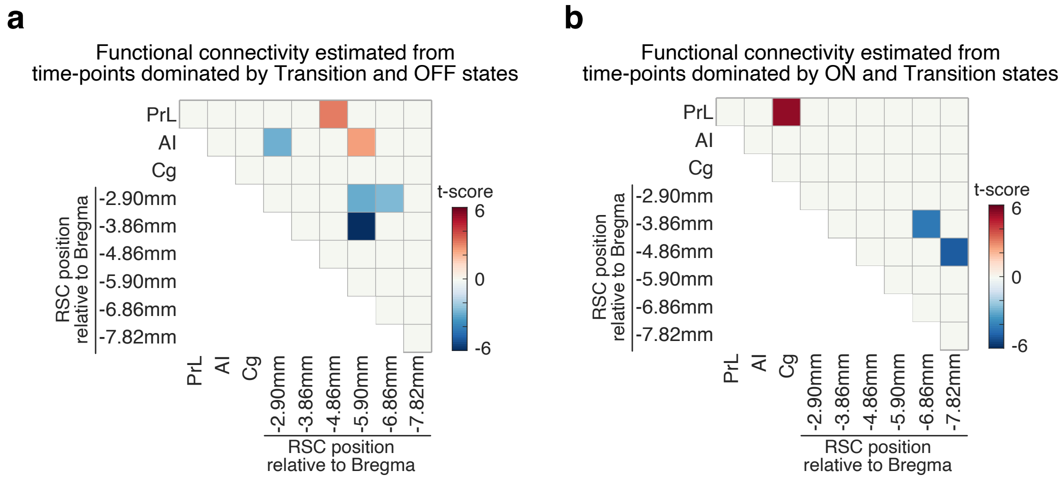

Figure S10. Replication of dynamic functional connectivity changes during the Transition compared to the ON and OFF states. (a) Comparison of functional connectivity between Transition and OFF states. **(b)** Comparison of functional connectivity between ON and Transition states. **(a, b)** All *ps* < 0.05, determined by two-tailed t-test with FDR correction; exact *p*-values provided in Source Data; $n = 9$ rats. Abbreviations: prelimbic cortex (PrL), anterior insular cortex (AI), cingulate cortex (Cg), retrosplenial cortex (RSC). These results converge on and validate functional connectivity changes reported in Figures 6c and 6f. Source data are provided as a Source Data file.

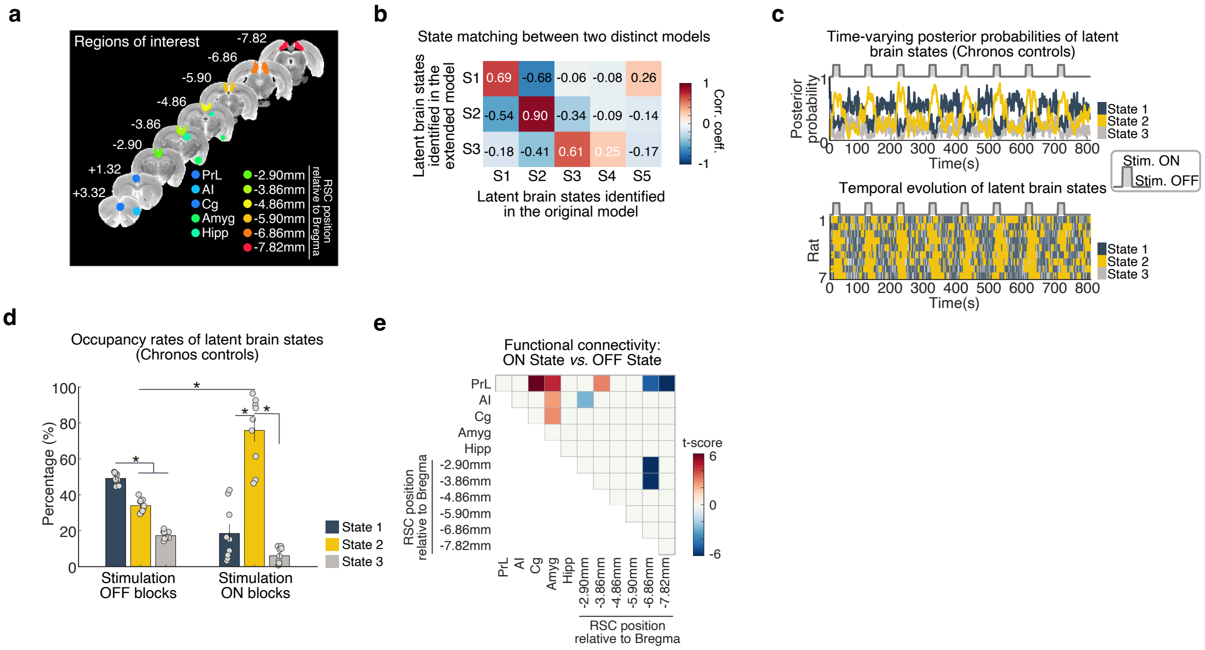

Figure S11. Replication of findings using extended salience network (SN) and default mode network (DMN) regions of interest (ROIs). (a) ROIs used in the extended model, including: prelimbic cortex (PrL), anterior insular cortex (AI), cingulate cortex (Cg), amygdala (Amyg), hippocampus (Hipp), and six retrosplenial cortex (RSC) subdivisions. **(b)** Correlation of posterior probabilities of latent brain states estimated from the original 9 ROI model and 11 ROI extended model. **(c)** Averaged time-varying posterior probabilities of the brain states identified in Chronos rats by the Bayesian switching dynamical systems (BSDS) model across the AI stimulation protocol (top). Temporal evolution of the brain states identified in Chronos rats (bottom). **(d)** Occupancy rates of the latent brain states in Chronos rats. Data are presented as mean ± SEM ($n = 9$ rats). *$p < 0.05$, p-values determined by two-tailed t-test, with FDR-correction; all exact p-values are provided in Source Data. **(e)** Specific links that showed significant differences in functional connectivity between the stimulation ON and OFF states (all $ps < 0.05$, determined by two-tailed t-test with FDR correction; exact p-values provided in Source Data; $n = 9$ rats). Source data are provided as a Source Data file.

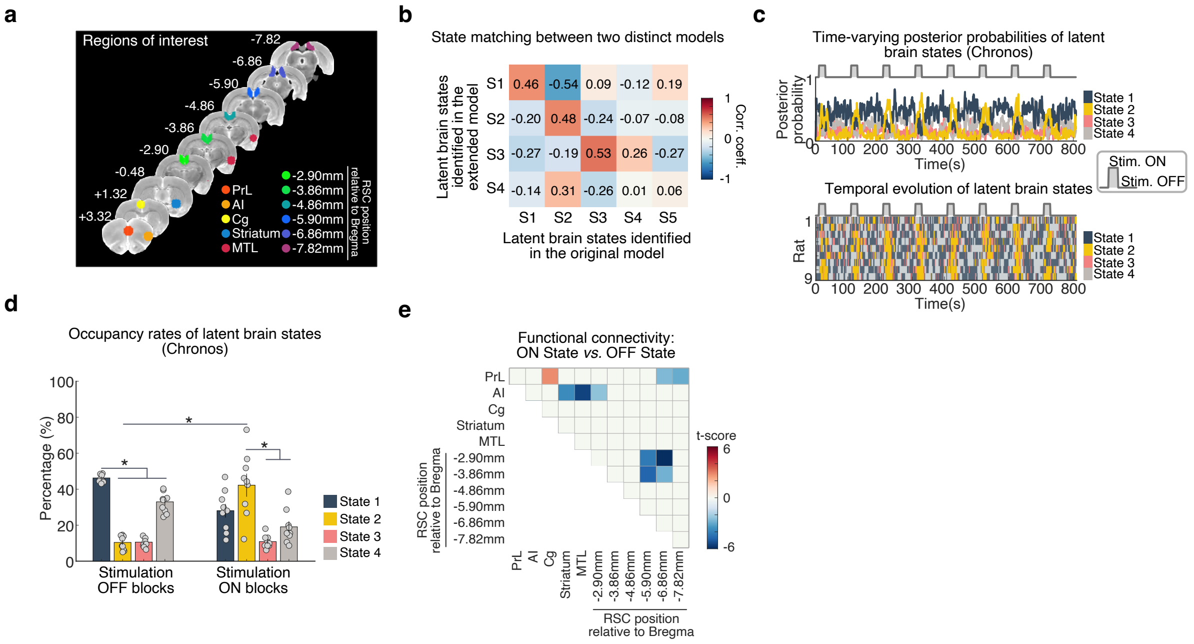

Figure S12. Replication of findings using salience network (SN) and default mode network (DMN) nodes and additional striatum-medial temporal lobe (MTL) regions of interest (ROIs). (a) ROIs used in the extended model, including: prelimbic cortex (PrL), anterior insular cortex (AI), cingulate cortex (Cg), striatum, MTL, and six retrosplenial cortex (RSC) subdivisions. **(b)** Correlation of posterior probabilities of latent brain states estimated from the original 9 ROI model and 11 ROI extended model. **(c)** Averaged time-varying posterior probabilities of the brain states identified in Chronos rats by the Bayesian switching dynamical systems (BSDS) model across the AI stimulation protocol (top). Temporal evolution of the brain states identified in Chronos rats (bottom). **(d)** Occupancy rates of the latent brain states in Chronos rats. Data are presented as mean ± SEM ($n = 9$ rats). *$p < 0.05$, p-values determined by two-tailed t-test, with FDR-correction; all exact p-values are provided in Source Data. **(e)** Specific links that showed significant differences in functional connectivity between the stimulation ON and OFF states (all $ps < 0.05$, determined by two-tailed t-test with FDR correction; exact p-values provided in Source Data; $n = 9$ rats). Source data are provided as a Source Data file.

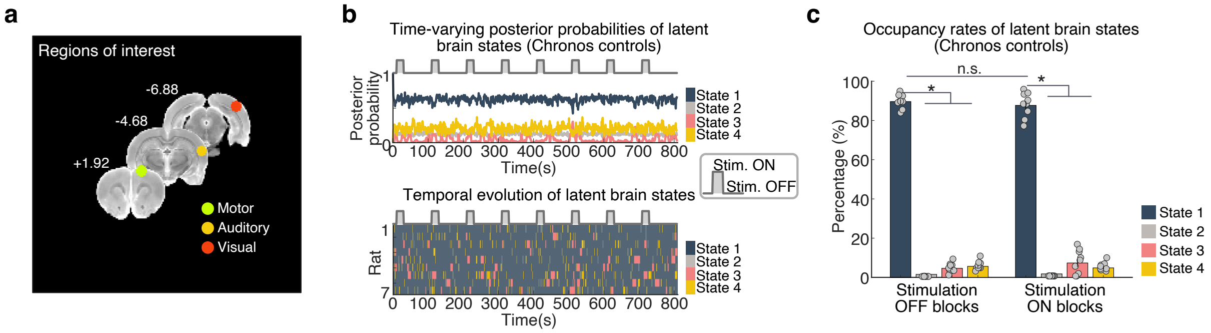

Figure S13. Control analysis using regions of interest (ROIs) located outside the default mode network (DMN) and salience network (SN). (a) ROIs used in control analysis. **(b)** Averaged time-varying posterior probabilities of the brain states identified in Chronos rats by the Bayesian switching dynamical systems (BSDS) model across the anterior insular cortex (AI) stimulation protocol (top). Temporal evolution of the brain states identified in Chronos rats (bottom). **(c)** Occupancy rates of the latent brain states in Chronos rats. State 1 dominated both stimulation ON and stimulation OFF blocks. Data are presented as mean ± SEM ($n = 9$ rats). *$p < 0.05$, p-values determined by two-tailed t-test, with FDR-correction; all exact p-values are provided in Source Data. Source data are provided as a Source Data file.

References

1. Taghia J, *et al.* Uncovering hidden brain state dynamics that regulate performance and decision-making during cognition. *Nature communications* **9**, 1-19 (2018).

2. Everett B. *An introduction to latent variable models*. Springer Science & Business Media (2013).

3. Ghahramani Z, Hinton GE. The EM algorithm for mixtures of factor analyzers.). Technical Report CRG-TR-96-1, University of Toronto (1996).

4. Fox EB. Bayesian nonparametric learning of complex dynamical phenomena.). Massachusetts Institute of Technology (2009).

5. Chang C-C, Lin C-J. LIBSVM: a library for support vector machines. *ACM transactions on intelligent systems and technology (TIST)* **2**, 1-27 (2011).

6. Cox RW. AFNI: software for analysis and visualization of functional magnetic resonance neuroimages. *Computers and Biomedical research* **29**, 162-173 (1996).

7. Leite FP, *et al.* Repeated fMRI using iron oxide contrast agent in awake, behaving macaques at 3 Tesla. *Neuroimage* **16**, 283-294 (2002).

8. McLaren DG, Ries ML, Xu G, Johnson SC. A generalized form of context-dependent psychophysiological interactions (gPPI): a comparison to standard approaches. *Neuroimage* **61**, 1277-1286 (2012).

9. Gogolla N. The insular cortex. *Current Biology* **27**, R580-R586 (2017).

10. Gehrlach DA, *et al.* A whole-brain connectivity map of mouse insular cortex. *eLife* **9**, e55585 (2020).

11. Nieuwenhuis B, *et al.* Optimization of adeno-associated viral vector-mediated transduction of the corticospinal tract: comparison of four promoters. *Gene Therapy* **28**, 56-74 (2021).

12. Watakabe A, *et al.* Comparative analyses of adeno-associated viral vector serotypes 1, 2, 5, 8 and 9 in marmoset, mouse and macaque cerebral cortex. *Neuroscience Research* **93**, 144-157 (2015).

13. Nathanson JL, Yanagawa Y, Obata K, Callaway EM. Preferential labeling of inhibitory and excitatory cortical neurons by endogenous tropism of adeno-associated virus and lentivirus vectors. *Neuroscience* **161**, 441-450 (2009).

14. Anikeeva P, *et al.* Optetrode: a multichannel readout for optogenetic control in freely moving mice. *Nat Neurosci* **15**, 163-170 (2011).

15. Ryali S, *et al.* Combining optogenetic stimulation and fMRI to validate a multivariate dynamical systems model for estimating causal brain interactions. *Neuroimage* **132**, 398-405 (2016).

16. Cai W, Ryali S, Pasumarthy R, Talasila V, Menon V. Dynamic causal brain circuits during working memory and their functional controllability. *Nature communications* **12**, 1-16 (2021).

17. Ryali S, Supekar K, Chen T, Menon V. Multivariate dynamical systems models for estimating causal interactions in fMRI. *Neuroimage* **54**, 807-823 (2011).

18. Ryali S*, et al.* Multivariate dynamical systems-based estimation of causal brain interactions in fMRI: Group-level validation using benchmark data, neurophysiological models and human connectome project data. *J Neurosci Methods* **268**, 142-153 (2016).

19. Tsai P-J*, et al.* Converging structural and functional evidence for a rat salience network. *Biological Psychiatry* **88**, 867-878 (2020).

20. Lu H, Zou Q, Gu H, Raichle ME, Stein EA, Yang Y. Rat brains also have a default mode network. *Proc Natl Acad Sci U S A* **109**, 3979-3984 (2012).

21. Lee S-H*, et al.* An isotropic EPI database and analytical pipelines for rat brain resting-state fMRI. *Neuroimage* **243**, 118541 (2021).

22. Mandino F*, et al.* A triple-network organization for the mouse brain. *Molecular Psychiatry*, 1-8 (2021).

23. Upadhyay J*, et al.* Default-mode-like network activation in awake rodents. *PloS one* **6**, e27839 (2011).

24. Zerbi V, Grandjean J, Rudin M, Wenderoth N. Mapping the mouse brain with rs-fMRI: An optimized pipeline for functional network identification. *Neuroimage* **123**, 11-21 (2015).

25. Lu H, Zou Q, Gu H, Raichle ME, Stein EA, Yang Y. Rat brains also have a default mode network. *Proceedings of the National Academy of Sciences* **109**, 3979-3984 (2012).

26. Sridharan D, Levitin DJ, Menon V. A critical role for the right fronto-insular cortex in switching between central-executive and default-mode networks. *Proceedings of the National Academy of Sciences* **105**, 12569-12574 (2008).

27. Cottam WJ, Iwabuchi SJ, Drabek MM, Reckziegel D, Auer DP. Altered connectivity of the right anterior insula drives the pain connectome changes in chronic knee osteoarthritis. *Pain* **159**, 929 (2018).

28. Goulden N*, et al.* The salience network is responsible for switching between the default mode network and the central executive network: replication from DCM. *Neuroimage* **99**, 180-190 (2014).

29. Menon V, Uddin LQ. Saliency, switching, attention and control: a network model of insula function. *Brain structure and function* **214**, 655-667 (2010).

30. Qadir H, Krimmel SR, Mu C, Poulopoulos A, Seminowicz DA, Mathur BN. Structural connectivity of the anterior cingulate cortex, claustrum, and the anterior insula of the mouse. *Frontiers in neuroanatomy* **12**, 100 (2018).

31. Semedo J, Zandvakili A, Kohn A, Machens CK, Byron MY. Extracting latent structure from multiple interacting neural populations. In: *Advances in neural information processing systems*) (2014).

32. Glaser JI, Whiteway MR, Cunningham JP, Paninski L, Linderman SW. Recurrent switching dynamical systems models for multiple interacting neural populations. *bioRxiv*, (2020).

33. Lee B*, et al.* Latent brain state dynamics and cognitive flexibility in older adults. *Progress in Neurobiology*, 102180 (2021).

34. Cai W, Warren SL, Duberg K, Pennington B, Hinshaw SP, Menon V. Latent brain state dynamics distinguish behavioral variability, impaired decision-making, and inattention. *Molecular Psychiatry*, 1-14 (2021).

35. Cai W, Chen T, Szegletes L, Supekar K, Menon V. Aberrant time-varying cross-network interactions in children with attention-deficit/hyperactivity disorder and the relation to attention deficits. *Biological Psychiatry: Cognitive Neuroscience and Neuroimaging* **3**, 263-273 (2018).

36. Rashid B*, et al.* Classification of schizophrenia and bipolar patients using static and dynamic resting-state fMRI brain connectivity. *Neuroimage* **134**, 645-657 (2016).

37. Allen EA, Damaraju E, Plis SM, Erhardt EB, Eichele T, Calhoun VD. Tracking whole-brain connectivity dynamics in the resting state. *Cerebral cortex* **24**, 663-676 (2014).

38. Hutchison RM*, et al.* Dynamic functional connectivity: promise, issues, and interpretations. *Neuroimage* **80**, 360-378 (2013).

39. Taghia J, Ryali S, Chen T, Supekar K, Cai W, Menon V. Bayesian switching factor analysis for estimating time-varying functional connectivity in fMRI. *Neuroimage* **155**, 271-290 (2017).

40. Vidaurre D, Smith SM, Woolrich MW. Brain network dynamics are hierarchically organized in time. *Proceedings of the National Academy of Sciences* **114**, 12827-12832 (2017).

41. Vidaurre D, Quinn AJ, Baker AP, Dupret D, Tejero-Cantero A, Woolrich MW. Spectrally resolved fast transient brain states in electrophysiological data. *Neuroimage* **126**, 81-95 (2016).

42. Ryali S*, et al.* Temporal Dynamics and Developmental Maturation of Salience, Default and Central-Executive Network Interactions Revealed by Variational Bayes Hidden Markov Modeling. *PLoS Comput Biol* **12**, e1005138 (2016).

43. Trask S, Pullins SE, Ferrara NC, Helmstetter FJ. The anterior retrosplenial cortex encodes event-related information and the posterior retrosplenial cortex encodes context-related information during memory formation. *Neuropsychopharmacology*, 1-7 (2021).

44. Stafford JM*, et al.* Large-scale topology and the default mode network in the mouse connectome. *Proceedings of the National Academy of Sciences* **111**, 18745-18750 (2014).

45. Pagani M*, et al.* mTOR-related synaptic pathology causes autism spectrum disorder-associated functional hyperconnectivity. *Nature communications* **12**, 1-15 (2021).

46. Coletta L, Pagani M, Whitesell JD, Harris JA, Bernhardt B, Gozzi A. Network structure of the mouse brain connectome with voxel resolution. *Science Advances* **6**, eabb7187 (2020).

47. Whitesell JD*, et al.* Regional, Layer, and Cell-Type-Specific Connectivity of the Mouse Default Mode Network. *Neuron* **109**, 545-559. e548 (2021).

48. Sugar J, Witter MP, van Strien N, Cappaert N. The retrosplenial cortex: intrinsic connectivity and connections with the (para) hippocampal region in the rat. An interactive connectome. *Frontiers in neuroinformatics* **5**, 7 (2011).

49. Grandjean J, Zerbi V, Balsters JH, Wenderoth N, Rudin M. Structural basis of large-scale functional connectivity in the mouse. *Journal of Neuroscience* **37**, 8092-8101 (2017).

50. Tu W, Ma Z, Ma Y, Dopfel D, Zhang N. Suppressing anterior cingulate cortex modulates default mode network and behavior in awake rats. *Cerebral cortex* **31**, 312-323 (2021).

51. Arsenault JT, Caspari N, Vandenberghe R, Vanduffel W. Attention shifts recruit the monkey default mode network. *Journal of Neuroscience* **38**, 1202-1217 (2018).

52. Hsu L-M*, et al.* Constituents and functional implications of the rat default mode network. *Proceedings of the National Academy of Sciences* **113**, E4541-E4547 (2016).

53. Mantini D*, et al.* Default mode of brain function in monkeys. *Journal of Neuroscience* **31**, 12954-12962 (2011).

54. Gozzi A, Schwarz AJ. Large-scale functional connectivity networks in the rodent brain. *Neuroimage* **127**, 496-509 (2016).

55. Seamans JK, Lapish CC, Durstewitz D. Comparing the prefrontal cortex of rats and primates: insights from electrophysiology. *Neurotox Res* **14**, 249-262 (2008).

56. Yang S-T, Shi Y, Wang Q, Peng J-Y, Li B-M. Neuronal representation of working memory in the medial prefrontal cortex of rats. *Molecular Brain* **7**, 61 (2014).

57. Paxinos G, Watson C. The rat brain in stereotaxic coordinates sixth edition by. *Acad Press* **170**, 10.1016 (2006).

58. Datta D*, et al.* Glutamate Carboxypeptidase II in Aging Rat Prefrontal Cortex Impairs Working Memory Performance. *Front Aging Neurosci* **13**, 760270 (2021).

59. Ramos BP, Colgan L, Nou E, Ovadia S, Wilson SR, Arnsten AFT. The Beta-1 Adrenergic Antagonist, Betaxolol, Improves Working Memory Performance in Rats and Monkeys. *Biological Psychiatry* **58**, 894-900 (2005).

60. Wang M*, et al.* α2A-Adrenoceptors Strengthen Working Memory Networks by Inhibiting cAMP-HCN Channel Signaling in Prefrontal Cortex. *Cell* **129**, 397-410 (2007).

61. Zahrt J, Taylor JR, Mathew RG, Arnsten AFT. Supranormal Stimulation of D₁ Dopamine Receptors in the Rodent Prefrontal Cortex Impairs Spatial Working Memory Performance. *The Journal of Neuroscience* **17**, 8528-8535 (1997).

62. Yang ST, Shi Y, Wang Q, Peng JY, Li BM. Neuronal representation of working memory in the medial prefrontal cortex of rats. *Mol Brain* **7**, 61 (2014).

63. Vogel P, Hahn J, Duvarci S, Sigurdsson T. Prefrontal pyramidal neurons are critical for all phases of working memory. *Cell Reports* **39**, 110659 (2022).

64. Aguilar DD, McNally JM. Subcortical control of the default mode network: Role of the basal forebrain and implications for neuropsychiatric disorders. *Brain Research Bulletin* **185**, 129-139 (2022).

65. Nair J, Klaassen A-L, Arato J, Vyssotski AL, Harvey M, Rainer G. Basal forebrain contributes to default mode network regulation. *Proceedings of the National Academy of Sciences* **115**, 1352-1357 (2018).

66. Klaassen A-L, Heiniger A, Vaca Sánchez P, Harvey MA, Rainer G. Ventral pallidum regulates the default mode network, controlling transitions between internally and externally guided behavior. *Proceedings of the National Academy of Sciences* **118**, e2103642118 (2021).

67. Lozano-Montes L*, et al.* Optogenetic Stimulation of Basal Forebrain Parvalbumin Neurons Activates the Default Mode Network and Associated Behaviors. *Cell Reports* **33**, 108359 (2020).

68. McNally JM*, et al.* Optogenetic manipulation of an ascending arousal system tunes cortical broadband gamma power and reveals functional deficits relevant to schizophrenia. *Molecular Psychiatry* **26**, 3461-3475 (2021).

69. Espinosa N, Alonso A, Lara-Vasquez A, Fuentealba P. Basal forebrain somatostatin cells differentially regulate local gamma oscillations and functionally segregate motor and cognitive circuits. *Scientific Reports* **9**, 2570 (2019).

70. Espinosa N, Alonso A, Morales C, Espinosa P, Chávez AE, Fuentealba P. Basal Forebrain Gating by Somatostatin Neurons Drives Prefrontal Cortical Activity. *Cerebral Cortex* **29**, 42-53 (2017).

71. Peeters LM, van den Berg M, Hinz R, Majumdar G, Pintelon I, Keliris GA. Cholinergic Modulation of the Default Mode Like Network in Rats. *iScience* **23**, 101455 (2020).

72. Oyarzabal EA*, et al.* Chemogenetic stimulation of tonic locus coeruleus activity strengthens the default mode network. *Sci Adv* **8**, eabm9898 (2022).

73. Do JP*, et al.* Cell type-specific long-range connections of basal forebrain circuit. *eLife* **5**, e13214 (2016).

74. Gompf HS*, et al.* Locus ceruleus and anterior cingulate cortex sustain wakefulness in a novel environment. *J Neurosci* **30**, 14543-14551 (2010).

75. Krieger-Redwood K*, et al.* Down but not out in posterior cingulate cortex: Deactivation yet functional coupling with prefrontal cortex during demanding semantic cognition. *NeuroImage* **141**, 366-377 (2016).

76. Stacho M, Manahan-Vaughan D. Mechanistic flexibility of the retrosplenial cortex enables its contribution to spatial cognition. *Trends in Neurosciences*, (2022).

77. Zingg B*, et al.* Neural networks of the mouse neocortex. *Cell* **156**, 1096-1111 (2014).

78. van Groen T, Michael Wyss J. Connections of the retrosplenial granular a cortex in the rat. *Journal of Comparative Neurology* **300**, 593-606 (1990).

79. van Groen T, Wyss JM. Connections of the retrosplenial dysgranular cortex in the rat. *Journal of Comparative Neurology* **315**, 200-216 (1992).

80. Van Groen T, Wyss JM. Connections of the retrosplenial granular b cortex in the rat. *Journal of Comparative Neurology* **463**, 249-263 (2003).

81. Chrastil ER. Heterogeneity in human retrosplenial cortex: A review of function and connectivity. *Behav Neurosci* **132**, 317-338 (2018).

82. Mandino F*, et al.* Animal Functional Magnetic Resonance Imaging: Trends and Path Toward Standardization. *Front Neuroinform* **13**, 78 (2019).

83. Pais-Roldán P*, et al.* Contribution of animal models toward understanding resting state functional connectivity. *Neuroimage* **245**, 118630 (2021).

84. Fukuda M, Vazquez AL, Zong X, Kim S-G. Effects of the α2-adrenergic receptor agonist dexmedetomidine on neural, vascular and BOLD fMRI responses in the somatosensory cortex. *European Journal of Neuroscience* **37**, 80-95 (2013).

85. Lee SH*, et al.* An isotropic EPI database and analytical pipelines for rat brain resting-state fMRI. *Neuroimage* **243**, 118541 (2021).

86. Paasonen J, Stenroos P, Salo RA, Kiviniemi V, Gröhn O. Functional connectivity under six anesthesia protocols and the awake condition in rat brain. *NeuroImage* **172**, 9-20 (2018).

87. Reimann HM, Niendorf T. The (Un)Conscious Mouse as a Model for Human Brain Functions: Key Principles of Anesthesia and Their Impact on Translational Neuroimaging. *Front Syst Neurosci* **14**, 8 (2020).

88. Gutierrez-Barragan D*, et al.* Unique spatiotemporal fMRI dynamics in the awake mouse brain. *Current Biology* **32**, 631-644. e636 (2022).

89. Liang Z, Liu X, Zhang N. Dynamic resting state functional connectivity in awake and anesthetized rodents. *Neuroimage* **104**, 89-99 (2015).

90. Bekhbat M, Merrill L, Kelly SD, Lee VK, Neigh GN. Brief anesthesia by isoflurane alters plasma corticosterone levels distinctly in male and female rats: Implications for tissue collection methods. *Behav Brain Res* **305**, 122-125 (2016).

91. Navarro KL, Huss M, Smith JC, Sharp P, Marx JO, Pacharinsak C. Mouse Anesthesia: The Art and Science. *ILAR Journal* **62**, 238-273 (2021).

92. Zambricki EA, Dalecy LG. Rat sex differences in anesthesia. *Comp Med* **54**, 49-53 (2004).

93. Sirmpilatze N, Baudewig J, Boretius S. Temporal stability of fMRI in medetomidine-anesthetized rats. *Scientific Reports* **9**, 16673 (2019).

94. Ernst M*, et al.* Pubertal maturation and sex effects on the default-mode network connectivity implicated in mood dysregulation. *Translational Psychiatry* **9**, 103 (2019).

95. Lawrence KE*, et al.* Sex Differences in Functional Connectivity of the Salience, Default Mode, and Central Executive Networks in Youth with ASD. *Cerebral Cortex* **30**, 5107-5120 (2020).

96. Mak LE, Minuzzi L, MacQueen G, Hall G, Kennedy SH, Milev R. The default mode network in healthy individuals: a systematic review and meta-analysis. *Brain connectivity* **7**, 25-33 (2017).

97. Conrin SD*, et al.* From default mode network to the basal configuration: sex differences in the resting-state brain connectivity as a function of age and their clinical correlates. *Frontiers in Psychiatry* **9**, 365 (2018).

98. Ficek-Tani B*, et al.* Sex differences in default mode network connectivity in healthy aging adults. *Cereb Cortex*, (2022).

99. Grissom NM, Reyes TM. Let's call the whole thing off: evaluating gender and sex differences in executive function. *Neuropsychopharmacology* **44**, 86-96 (2019).

100. Kilpatrick LA*, et al.* Sex commonalities and differences in the relationship between resilient personality and the intrinsic connectivity of the salience and default mode networks. *Biological Psychology* **112**, 107-115 (2015).

101. Dumais KM, Chernyak S, Nickerson LD, Janes AC. Sex differences in default mode and dorsal attention network engagement. *PLoS One* **13**, e0199049 (2018).

102. Long H*, et al.* Sex-related Difference in Mental Rotation Performance is Mediated by the special Functional Connectivity Between the Default Mode and Salience Networks. *Neuroscience* **478**, 65-74 (2021).

103. Fukuda M, Poplawsky AJ, Kim S-G. Time-dependent spatial specificity of high-resolution fMRI: insights into mesoscopic neurovascular coupling. *Philosophical Transactions of the Royal Society B* **376**, 20190623 (2021).

104. Schridde U, Khubchandani M, Motelow JE, Sanganahalli BG, Hyder F, Blumenfeld H. Negative BOLD with large increases in neuronal activity. *Cerebral cortex* **18**, 1814-1827 (2008).
