# Supplementary material for: Optogenetic stimulation of anterior insular cortex neurons in male rats reveals causal mechanisms underlying suppression of the default mode network by the salience network
Source: Nat Commun. 2023 Feb 16;14:866. doi: 10.1038/s41467-023-36616-8 (PMC9935890; doi:10.1038/s41467-023-36616-8)
Supplement: Supplementary file 3 — Reporting Summary [file 41467_2023_36616_MOESM3_ESM.pdf]

nature portfolio

Corresponding author(s): Vinod Menon, Yen-Yu Ian Shih

Last updated by author(s): Feb 6, 2023

Reporting Summary

Nature Portfolio wishes to improve the reproducibility of the work that we publish. This form provides structure for consistency and transparency in reporting. For further information on Nature Portfolio policies, see our Editorial Policies and the Editorial Policy Checklist.

Statistics

For all statistical analyses, confirm that the following items are present in the figure legend, table legend, main text, or Methods section.

n/a	Confirmed	
☐	☒	The exact sample size (n) for each experimental group/condition, given as a discrete number and unit of measurement
☐	☒	A statement on whether measurements were taken from distinct samples or whether the same sample was measured repeatedly
☐	☒	The statistical test(s) used AND whether they are one- or two-sided<br>*Only common tests should be described solely by name; describe more complex techniques in the Methods section.*
☐	☒	A description of all covariates tested
☐	☒	A description of any assumptions or corrections, such as tests of normality and adjustment for multiple comparisons
☐	☒	A full description of the statistical parameters including central tendency (e.g. means) or other basic estimates (e.g. regression coefficient) AND variation (e.g. standard deviation) or associated estimates of uncertainty (e.g. confidence intervals)
☐	☒	For null hypothesis testing, the test statistic (e.g. F, t, r) with confidence intervals, effect sizes, degrees of freedom and P value noted<br>*Give P values as exact values whenever suitable.*
☐	☒	For Bayesian analysis, information on the choice of priors and Markov chain Monte Carlo settings
☒	☐	For hierarchical and complex designs, identification of the appropriate level for tests and full reporting of outcomes
☐	☒	Estimates of effect sizes (e.g. Cohen's d, Pearson's r), indicating how they were calculated

Our web collection on statistics for biologists contains articles on many of the points above.

Software and code

Policy information about availability of computer code

Data collection	MRI data was acquired using the ParaVision 5 preclinical imaging software (Bruker Corporation). MRI data was preprocessed using the open source Analysis of Functional NeuroImages (AFNI Ver.20.2.10) software suite. Optogenetic stimulus triggers were delivered according the the stimulation paradigm and synchronized to fMRI acquisition, via a DAQ board controlled by a homemade software program, PawStim.
Data analysis	Matlab (R2019b) was used for all the data analysis. Stimulation block classification analysis was performed using the MATLAB package LIBSVM (v.3.3). We have provided a Zenodo web-link for the custom code used in this study: https://zenodo.org/badge/latestdoi/434680845.

For manuscripts utilizing custom algorithms or software that are central to the research but not yet described in published literature, software must be made available to editors and reviewers. We strongly encourage code deposition in a community repository (e.g. GitHub). See the Nature Portfolio guidelines for submitting code & software for further information.

Data

Policy information about availability of data

All manuscripts must include a data availability statement. This statement should provide the following information, where applicable:

- Accession codes, unique identifiers, or web links for publicly available datasets
- A description of any restrictions on data availability
- For clinical datasets or third party data, please ensure that the statement adheres to our policy

All original data reported in this study are publicly available on Zenodo: https://zenodo.org/badge/latestdoi/434680845. Source data are provided with this paper.

Human research participants

Policy information about studies involving human research participants and Sex and Gender in Research.

Reporting on sex and gender	This study does not involve human research participants.
Population characteristics	This study does not involve human research participants.
Recruitment	This study does not involve human research participants.
Ethics oversight	This study does not involve human research participants.

Note that full information on the approval of the study protocol must also be provided in the manuscript.

Field-specific reporting

Please select the one below that is the best fit for your research. If you are not sure, read the appropriate sections before making your selection.

☒ Life sciences ☐ Behavioural & social sciences ☐ Ecological, evolutionary & environmental sciences

For a reference copy of the document with all sections, see nature.com/documents/nr-reporting-summary-flat.pdf

Life sciences study design

All studies must disclose on these points even when the disclosure is negative.

Sample size	The sample size chosen for this study was above average (not considering group distribution) for rat fMRI studies as a whole (https://doi.org/10.3389/fninf.2019.00078), and exceeded that of our previously published studies using optogenetics-fMRI (https://doi.org/10.1038/srep31613 , https://doi.org/10.1016%2Fj.neuroimage.2016.02.067).
Data exclusions	No data were excluded from the analyses.
Replication	To promote reproducibility, all data and analyses used in this study have been made publicly available on Zenodo: https://zenodo.org/badge/latestdoi/434680845.
Randomization	Subjects were randomly assigned to experimental or control groups at the time of surgical preparation.
Blinding	Subjects were labelled with a numeric animal ID at group assignment, and this ID was the only identifier used for data acquisition during fMRI scanning and histological verification of stimulation locations. Group assignment, animal surgery and fMRI scanning were carried out by one individual, histology, data preprocessing and analysis by others.

Reporting for specific materials, systems and methods

We require information from authors about some types of materials, experimental systems and methods used in many studies. Here, indicate whether each material, system or method listed is relevant to your study. If you are not sure if a list item applies to your research, read the appropriate section before selecting a response.

Materials & experimental systems

n/a	Involved in the study	
☒	☐	Antibodies
☒	☐	Eukaryotic cell lines
☒	☐	Palaeontology and archaeology
☐	☒	Animals and other organisms
☒	☐	Clinical data
☒	☐	Dual use research of concern

Methods

n/a	Involved in the study	
☒	☐	ChIP-seq
☒	☐	Flow cytometry
☐	☒	MRI-based neuroimaging

Animals and other research organisms

Policy information about studies involving animals; ARRIVE guidelines recommended for reporting animal research, and Sex and Gender in Research

Laboratory animals	This study used Sprague Dawley rats (Charles River Labs), ~60 days old and weighing ~300g at the time of surgery.
Wild animals	This study did not involve wild animals.
Reporting on sex	Sex was not considered in the design of this study. All rats used herein were assigned as male by the supplier, Charles River Labs.
Field-collected samples	This study did not involve samples collected from the field.
Ethics oversight	All animal procedures were performed in strict accordance with the National Institutes of Health Guidelines for Animal Research (Guide for the Care and Use of Laboratory Animals, eight edition) and reviewed and approved by the University of North Carolina Institutional Animal Care and Use Committee (protocol #15-057.0).

Note that full information on the approval of the study protocol must also be provided in the manuscript.

Magnetic resonance imaging

Experimental design

Design type	Optogenetic stimulation was delivered in a repeated epoch design.
Design specifications	Stimulation was delivered in a repeated epoch design, consisting of 8 repetitions of a 20 s "ON" block of pulsed 473 nm blue-light delivery via the chronically-implanted optical fiber in AI followed by an 80 s "OFF" block without stimulation to allow recovery of neuronal activity to baseline.
Behavioral performance measures	No behavioral performance measure was used in this study.

Acquisition

Imaging type(s)	Functional, anatomical
Field strength	9.4-Tesla
Sequence & imaging parameters	fMRI scans were acquired with a single shot, gradient echo-EPI sequence optimized for the Feraheme CBV contrast agent with the following parameters: spectral width = 300 kHz, TR/TE = 1000/8 ms, FOV = 2.56 x 2.56 cm2, matrix size = 80x80. Anatomical images were acquired using a T2-weighted RARE sequence with the following parameters: spectral width: 47 kHz, TR/TE = 2500/33 ms, FOV = 2.56 x 2.56 cm2, matrix size = 256 x 256, RARE factor = 8, averages = 8. The initial Feraheme infusion fMRI scan was 300 s in duration and included a minimum of 60 s before and after the infusion to acquire the pre- and post-Feraheme image intensities, respectively.
Area of acquisition	All MRI acquisitions were in anisotropic resolution with 12 coronal slices, 1 mm thick and 1 mm apart, aligned on the AP axis such that the fifth-most anterior slice was centered on the anterior commissure (corresponding to -0.36 mm AP) for each rat.

Diffusion MRI ☐ Used ☒ Not used

Preprocessing

Preprocessing software	All MRI images were preprocessed using AFNI.
Normalization	A group template based on individual T2-weighted images was generated using procedures described by Kazemi et al. Individual subject fMRI data were then slice-timing corrected and realigned to the mean image to correct head-motion. Six degree of freedom motion parameters were estimated through the realignment step. Aligned images were then co-registered to the T2-weighted anatomical scan followed by spatially normalization to the T2-weighted group template using linear affine registration.
Normalization template	A group template based on individual T2-weighted images was generated using procedures described by Kazemi et al. Individual subject fMRI data were then slice-timing corrected and realigned to the mean image to correct head-motion. Six degree of freedom motion parameters were estimated through the realignment step. Aligned images were then co-registered to the T2-weighted anatomical scan followed by spatially normalization to the T2-weighted group template using linear affine registration.
Noise and artifact removal	The normalized functional images were resampled from 0.32 x 0.32 x 1 mm voxel size to 0.5 mm isotropic voxel size. Nuisance removal including detrending with 3rd order polynomial fitting, a high-pass filter with frequency cut at 0.01 Hz and regressing out the head motion parameters. Finally, a 1 mm FWHM Gaussian kernel was applied for spatial smoothing.
Volume censoring	Volume censoring was not used in this study.

Statistical modeling & inference

Model type and settings	We used a conventional general linear model (3dDeconvolve) as implemented in AFNI to determine subject-level brain activation maps and t-tests to determine group-level effects.<br><br>We used 'Bayesian Switching Dynamic Systems' model, which is based on a hidden Markov process model, to uncover latent brain states associated with optogenetic stimulation. We used a variational Bayesian framework to infer model parameters, including the number of brain states.
Effect(s) tested	Separate one sample t-tests were used to determine group-level activation maps to the optogenetic stimulation paradigm.

Specify type of analysis: ☐ Whole brain ☐ ROI-based ☒ Both

Anatomical location(s)	ROIs were determined from the rodent default-mode network and salience network literature and were set as 1mm spheres centered at the chosen anatomical locations. Anatomical locations was determined based on the Paxinos and Watson rat brain atlas, 6th edition.

Statistic type for inference<br>(See Eklund et al. 2016)	We used a voxel-wise threshold of $p < 0.005$ with family-wise a cluster-correction threshold of $p < 0.01$ (cluster size = 38 voxels) to determine significant clusters in group-level brain activation maps.
Correction	The FWE cluster-correction threshold was determined using 3dclustsim in AFNI.

Models & analysis

n/a	Involved in the study
☐	☒ Functional and/or effective connectivity
☒	☐ Graph analysis
☐	☒ Multivariate modeling or predictive analysis

Functional and/or effective connectivity	Partial correlation was used to estimate functional connectivity
Multivariate modeling and predictive analysis	We used 'Bayesian Switching Dynamic Systems' model, which is based on a hidden Markov process model, to uncover latent brain states associated with optogenetic stimulation. We used a variational Bayesian framework to infer model parameters, including the number of brain states. The number of states is treated as a random variable, whose optimal value is learned from data using automatic relevance determination procedures implemented in a variational Bayesian framework. Detailed theoretical derivations and parameters are provided in our previous study (https://doi.org/10.1038/s41467-018-04723-6).
